# Soluble CD4 inhibits Ebola virus infection by targeting endosomal receptor-binding site

**DOI:** 10.1016/j.isci.2025.112573

**Published:** 2025-05-02

**Authors:** Leah Liu Wang, Patrick Keiser, Derek Yang, Javier Seravalli, J.J. Patten, Brett Eaton, Dirk Anderson, Yi Liu, Michael R. Holbrook, Amos B. Smith, Robert A. Davey, Shi-Hua Xiang

**Affiliations:** 1 School of Veterinary Medicine and Biomedical Sciences and Nebraska Center for Virology, University of Nebraska-Lincoln, Lincoln, NE 68583, USA; 2 National Emerging Infectious Diseases Laboratories, Boston University, Boston, MA 0211, USA; 3 Department of Chemistry, University of Pennsylvania, Philadelphia, PA 19104, USA; 4 Department of Biochemistry and Nebraska Center for Biotechnology, University of Nebraska-Lincoln, Lincoln, NE 68588, USA; 5 Integrated Research Facility at Fort Detrick, National Institute of Allergy and Infectious Diseases, Frederick, MD 21702, USA; 6 Holland Computing Center, University of Nebraska-Lincoln, Lincoln, NE 68588, USA

**Keywords:** Biological sciences, Immunology, Microbiology, Natural sciences, Virology

## Abstract

Human CD4 (cluster of differentiation 4) is well known as the primary receptor for human immunodeficiency virus (HIV) entry into the cells. The virus binds to CD4 molecules to induce a conformational change in the viral glycoprotein (GP) gp120, which exposes the co-receptor binding site for coreceptors CCR5 or CXCR4. The co-receptor binding then leads to membrane fusion for viral entry. Since the CD4 molecule has a high affinity for gp120, soluble CD4 (sCD4) and CD4-mimetic small molecules (CD4mcs) have been extensively studied as potential inhibitors for HIV infection. Surprisingly, we have found that human sCD4 and some CD4mcs are able to inhibit Ebola virus (EBOV) infection. Evidence is provided that the compounds block viral entry by targeting the GP binding site for the endosomal receptor Niemann-Pick C1 (NPC1). This finding reveals virus-receptor binding similarities between two remote viruses (HIV and EBOV) and suggests new possibilities for EBOV entry inhibitors.

## Highlights

• Soluble CD4 (sCD4) inhibits Ebola virus infection
• CD4-mimetic small compounds (CD4mcs) inhibit Ebola virus infection
• sCD4 and CD4mcs bind to NPC1 receptor binding site (RBS)
• sCD4 and CD4mcs inhibit EBOV infection by targeting NPC1 RBS

## Graphical abstract

**Figure:**
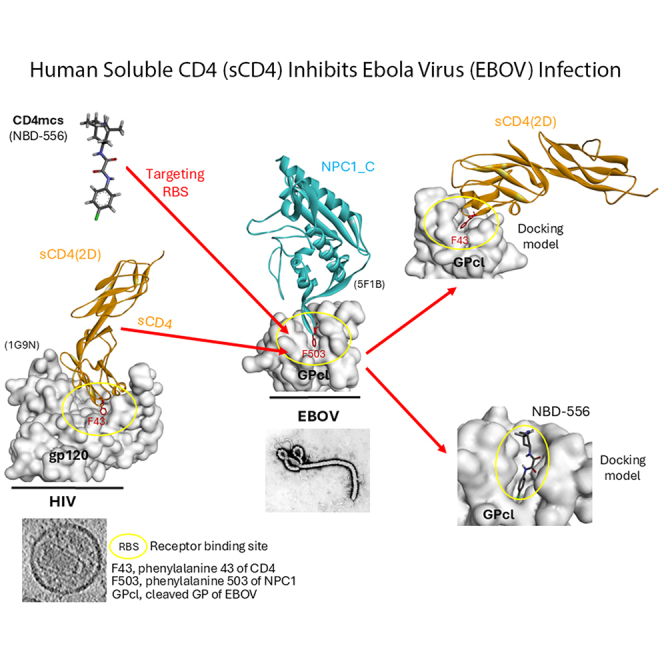

## Introduction

Human CD4 (cluster of differentiation 4) is an important membrane glycoprotein (GP) that serves as a coreceptor for T-cell receptor (TCR),^1^^,^^2^ and is also utilized by human immunodeficiency virus (HIV) as the primary receptor for infection.^3^^,^^4^^,^^5^ HIV infection occurs through binding to CD4, which triggers viral gp120 conformational changes to expose the binding site for the coreceptors, CCR5 or CXCR4.^6^^,^^7^^,^^8^ Coreceptor binding causes further conformational changes in gp120 and gp41, resulting in membrane fusion and ultimately in viral entry.^9^^,^^10^ The CD4-binding site (CD4bs) has been a principal target for drug development against HIV infection. Usage of soluble CD4 (sCD4) or CD4-mimetic small molecules (CD4mcs) to block HIV entry has been extensively studied in anti-HIV research.^11^^,^^12^^,^^13^^,^^14^^,^^15^^,^^16^^,^^17^^,^^18^ Unexpectedly, we have found that sCD4 and some CD4mcs, such as the prototypical compounds NBD-556 and NBD-557,^11^ have modest activities against Ebola virus (EBOV) infection.

EBOV belongs to the family Filoviridae*.* Infection by EBOV can lead to severe Ebola viral disease (EVD), which has a high mortality rate.^19^^,^^20^ Filoviruses are enveloped filamentous RNA viruses with a single-stranded negative-sense genome of approximately 19 kb.^21^^,^^22^ The envelope spike GP is the sole viral protein on the virion surface and is required for viral entry. The GP is primarily biosynthesized as a single polypeptide, which undergoes trimer formation, and it is later cleaved by a cellular protease (Furin) into the GP1 and GP2 subunits.^23^ The entry of EBOV occurs via macropinocytosis and trafficking into endosomes or lysosomes.^24^^,^^25^^,^^26^ In the late endosomes or lysosomes, GP1 is further cleaved by cathepsin proteases L and B that remove the glycan cap and the heavily glycosylated mucin-like domain (MLD), resulting in the exposure of the receptor binding site (RBS) for interacting with the endosomal receptor Nieman-Pick C1 (NPC1), a transporter of cholesterol.^27^^,^^28^ The cleaved GP (GPcl) that includes the GPcl1 and part of GP2 can bind to the endosomal receptor NPC1.^29^^,^^30^ This binding triggers a GP2 conformational change and leads to viral and cell membrane fusion that facilitates viral entry into the host cell cytoplasm.^31^^,^^32^ Therefore, targeting the RBS on GP could potentially prevent EBOV infection.

Although both HIV and EBOV have class I fusion GPs, sharing a common membrane fusion mechanism, they are quite different in sequence and receptor specificity. The interaction of HIV and receptor CD4 occurs on the cell surface, while the interaction of EBOV and receptor NPC1 occurs in the late endosomes or lysosomes under acidic conditions. The CD4bs^6^^,^^33^ of HIV gp120 is open and inducible, but the NPC1 binding site (NPC1bs)^29^^,^^30^ of EBOV-GP is not exposed and it is covered by a highly glycosylated cap domain that is cleaved off by endosomal proteases in the late endosomes or lysosomes for viral entry. However, we have found some structural parallels between the two RBSs of the two viruses that are exemplified by hydrophobic interactions involving protruding loop-based phenylalanine (Phe) residues.^6^^,^^30^ These distantly related viruses utilize loop-projecting phenyl-ring insertions within deep hydrophobic cavities to bind to their receptors. This finding holds substantial importance for the development of a novel class of drugs targeting the NPC1 RBS with the aim of the neutralizing EBOV.

## Results

### Soluble CD4 and CD4-derived proteins inhibit EBOV infection

**Figure 1:**
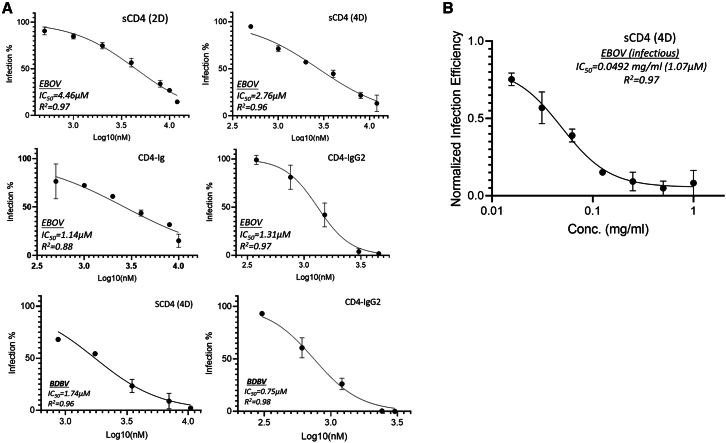
Inhibition assay of sCD4 and CD4-derived protein molecules against pseudotyped and infectious Ebola viruses (A) sCD4 (2D), soluble first two-domain CD4; sCD4 (4D), soluble four-domain CD4; CD4-Ig, first two-domain CD4 linked with human Fc region (Ig domain)^35^; CD4-IgG2, first two-domain CD4 with human Fc region (Ig domain); EBOV, Zaire Ebola virus; BDBV, Bundibugyo ebolavirus. All experiments were conducted in triplicates and the error bars represent standard deviations from three experiments. (B) sCD4 (4D) inhibits infectious Ebola virus infection. This experiment was conducted in BSL-4 containment in triplicates, and the error bars represent standard deviations.

Human sCD4 has four extracellular immunoglobulin-like domains with a molecular weight of ∼45 kD, where the first domain (D1) is responsible for the interaction with the CD4bs of HIV gp120. Additionally, the first two-domains of CD4 (2D-CD4)^34^ have been found to have similar binding affinity to the gp120 as four domain CD4 (4D-CD4) and has commonly been used in HIV research to study this interaction. Here, we used 4D-CD4 and 2D-CD4 to assess their activities against pseudotyped EBOV. Our results indicate that both 4D-CD4 and 2D-CD4 proteins can inhibit EBOV infection (Figure 1), but the 4D-CD4 (IC_50_, 2.76 μM) has a better activity than 2D-CD4 (IC_50_, 4.46 μM). Furthermore, we have also evaluated CD4-derived molecules such as CD4-Ig and CD4-IgG2, which were generated by fusion of 2D-CD4 to an antibody Ig domain (Fc region).^34^^,^^35^ CD4-Ig has two 2D-CD4 subunits fused to an Ig domain, whereas IgG2 has four 2D-CD4 subunits fused with an Ig domain. Both showed improved activities against EBOV infection, with IC_50_ values of 1.14 μM and 1.31 μM, respectively (Figure 1A). We also assessed another Ebolavirus species, Bundibugyo ebolavirus (BDBV), with similar outcomes to those of EBOV (Zaire EBOV). Specifically, sCD4 and CD4-IgG2 have IC_50_ values of 1.74 μM and 0.75 μM, respectively, against BDBV infection (Figure 1A), with CD4-IgG2 inhibitory activity for BDBV reaching sub-micromolar levels. To validate this finding using authentic infectious filoviruses, sCD4 (4D) was evaluated against the infectious EBOV virus at a BSL-4 containment facility. These studies demonstrated antiviral activity with an IC_50_ value of 1.07 μM (Figure 1B), which is comparable to the result of pseudotyped EBOV virus.

### CD4-mimetic small molecules inhibit EBOV infection

**Figure 2:**
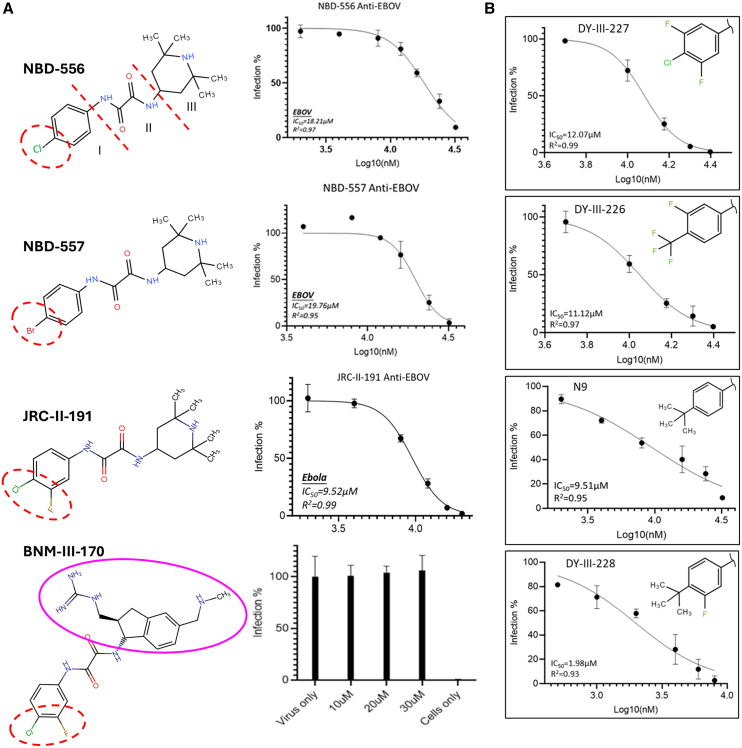
Inhibition assay of CD4-mimetic small molecules against pseudotyped Ebola viruses (A) NBD-556 and analogs inhibiting EBOV infection. (B) Newly designed and synthesized compounds with phenyl ring substituted molecules in the region-I inhibiting EBOV. All experiments were conducted in triplicates and the error bars represent standard deviations from three experiments.

CD4-mimetic small molecules (CD4mcs) are a class of small molecules that mirror the binding of natural CD4 at the HIV-1 gp120 RBS and ultimately interfere with the CD4-gp120 interaction necessary for viral entry. The prototypic CD4-mimetic compounds NBD-556 and NBD-557 were first identified in 2005^11^ (Figure 2A), and can be divided into three regions: region I (substituted phenyl ring), region II (oxalamide linker), and region III (tetramethyl piperidine).^36^^,^^37^ These two small molecules differ only in the substitutions on the phenyl ring where one is chloro (Cl) and another one is bromo (Br). Like sCD4 and CD4-derived protein molecules, NBD-556 and NBD-557 exhibited activities against EBOV infection with IC_50_ values of 18.21 μM and 19.76 μM, respectively (Figure 2A). We hypothesized that region I of these molecules operates similarly to CD4 and binds to the receptor NPC1-binding site (NPC1bs). Therefore, we assessed another CD4-mimetic compound, JRC-II-191, which has an additional fluoro (F) atom adjacent to the chloro (Cl) substituent of the phenyl ring in region I. This substitution resulted in an improvement of potency. By contrast, when region III was also changed to a bulkier group such as in BNM-III-170, the inhibition potency was largely reduced (Figure 2A). Other analogs altered in region III (Figure S1) also largely reduced or lost the activities against EBOV (data not shown). Therefore, it appears that region I is important for the CD4mc binding and region III changes negatively affect this activity. Following this hypothesis, we specifically designed three molecules, DY-III-226, DY-III-227, and DY-III-228, in which only region I was modified. We found that they all showed better activities, and especially DY-III-228 showed significantly improved potency against EBOV with an IC_50_ value of 1.98 μM (Figure 2B). The molecule resulting from the addition of a fluoro (F) substituent to compound N9, which has a *tert*-butyl at position 4 of the phenyl ring in region I, may potentially fit better in the binding cavity of the EBOV RBS. All these tested CD4mc compounds are listed and compared in Figure S1 and Table S1.

### Specificity test of sCD4 and CD4-mimetic compounds

**Figure 3:**
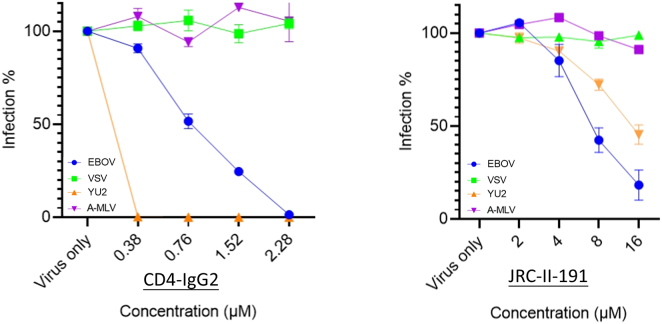
Specificity assay of CD4-IgG-2 and JRC-II-191 against pseudo-typed HIV, EBOV, VSV, and A-MLV CD4-mimetic molecules CD4-IgG2 and JRC-II-191 were evaluated against four different viruses: HIV (YU2), EBOV, VSV (vesicular stomatitis virus), and A-MLV (amphitropic murine leukemia virus). All experiments were conducted in triplicates and the error bars represent standard deviations from three experiments.

It was a surprise to find out that sCD4 and CD4mcs also have activities against filovirus infection. We then evaluated two other viruses commonly used as negative controls in antiviral research. One is the vesicular stomatitis virus (VSV), an enveloped, negative-sense RNA virus that infects a wide variety of mammalian and insect cells; another is the amphotropic murine leukemia virus (A-MLV), a retrovirus like HIV. We made pseudotyped viruses in the same manner as the EBOV pseudotypes by using an HIV-backbone. We evaluated the two better inhibitors for EBOV, CD4-IgG2 (protein molecule) and JRC-II-191 (small molecule), and found that these two inhibitors did not neutralize pseudotyped VSV or A-MLV (Figure 3). The results suggest that these antiviral activities of sCD4 and CD4mcs are specific for EBOV and HIV.

### sCD4 and CD4mc NBD-556 bind to EBOV GP-RBD

**Figure 4:**
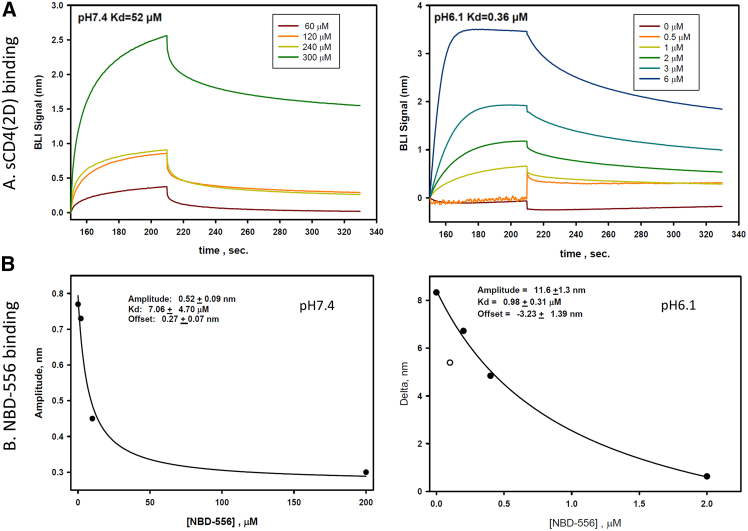
Binding affinity and kinetics assay of sCD4 (2D) and NBD-556 binding to EBOV receptor binding domain by biolayer interferometry Two-domain CD4 (sCD4-2D) (A) and NBD-556 (B) at two pH conditions, pH7.4 and pH6.1. Open circle in (B) was not included in the fit of the data. (See the STAR Methods BLI section for details).

To evaluate whether sCD4 binding to EBOV GP was direct, each protein was purified as sCD4 and GP-RBD-Fc (without the glycan cap and MLD) (Figure S2) and the interactions were monitored by bio-layer interferometry (BLI). The binding affinity of 2D-CD4 to GP-RBD was assessed at neutral pH (7.4) or acidic pH (6.1) (Figure 4A). The fitted K_D_ value is 52 μM at pH 7.4, contrasting with the much higher binding affinity of 0.36 μM at pH 6.1, as would be expected to be found in an endosome environment. The small molecule CD4mc NBD-556 was assayed in competition with sCD4 under these two different pH conditions and showed similar binding behavior. Under acidic conditions, the binding affinity is higher than under neutral pH conditions (Figure 4B). This observation underscores the requirement for endosomal acidic conditions in facilitating the NPC1-GP interaction for viral entry.^29^ However, a similar acidic pH-dependent phenomenon was noted in the binding affinity of sCD4 and EBOV-GP, a fact that needs to be further examined.

### sCD4 and CD4-mimetics compete with NPC1 receptor binding

**Figure 5:**
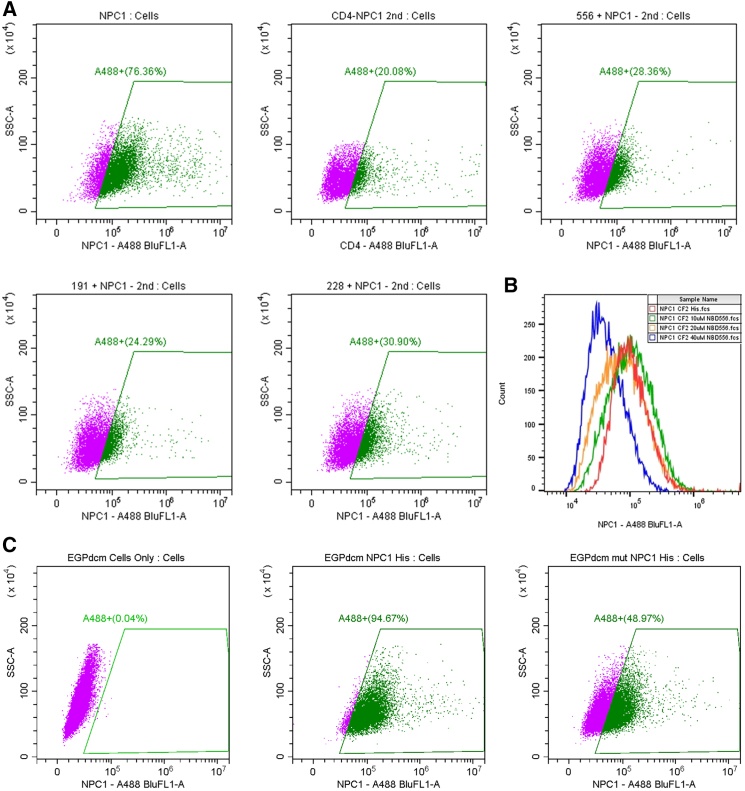
Binding competition assay of sCD4 and CD4-mimetic compounds with receptor NPC1 (A) sCD4, NBD-556, JRC-II-191, and DY-III-228 competing with NPC1 receptor. (B) Dose response (0, 10, 20, and 40 μM) of NBD-556 in binding competition with NPC1 receptor. (C) Comparison of wild-type (EGPDCM-WT) and mutant (EGPDCM-mut) (WF/AA, 86W/A, and 88F/A) receptor binding domain (RBD) of EBOV binding to the NPC1 receptor. The mutant (WF/AA) surface expression level was confirmed to be comparable to the wild type (see Figure S7). Representative of 3 experiments.

Since the EBOV GP-NPC1 interaction occurs within late endosomes or lysosomes, we developed a cell-surface binding model for examining ligand binding at the RBS on the cell surface and used it to confirm that sCD4 or CD4mcs inhibit filovirus infection by targeting the NPC1bs (Figure S3). EBOV-GP-RBDs without the glycan cap and MLD were displayed on the cell surface so ligand binding could be assessed. Unfortunately, the sCD4 direct binding assay signal yielded a background that was too high for directly monitoring of the binding. A viable alternative involves conducting a competition assay with the NPC1 receptor. sCD4 (2D-CD4) showed competition with NPC1 for binding to the Ebola GP-RBD; when a concentration of 20 μM sCD4 was used, the NPC1 binding reduced from 76.36% to 20.08% (Figure 5A). Consequently, the CD4mcs should also be able to compete with NPC1 binding and competition results indicated that indeed this was the case, as NBD-556 reduced NPC1 binding to 28.36% (Figure 5A). Additionally, the NBD-556 dose-response data (from 10 μM and 20 μM to 40 μM) further supported competition for the receptor NPC1 (Figure 5B). Other CD4mcs, JRC-II-191 and DY-III-228, showed similar competition effects with similar reduction rates (Figure 5A). To further confirm that sCD4 binds to the NPC1bs and competes with NPC1 binding, we mutated the RBS of EBOV-GP (EGPdcm) at positions W83A and F86A (WF/AA), both which are conserved in EBOV and BDBV and expected to be in direct contact for the interaction (Figure S4). These mutations of RBD (EGPdcm mut, WF/AA) largely reduced the NPC1 binding from 94.67% (WT) to 48.97% (mutant) (Figure 5C). The RBD mutant surface expression level was also confirmed to be comparable to the wild-type RBD (Figure S7). Thus, these data strongly suggest that all these tested compounds bind to the RBS of EBOV-GP and compete with receptor NPC1 binding.

### sCD4 and CD4-mimetics docked to the NPC1 RBS

**Figure 6:**
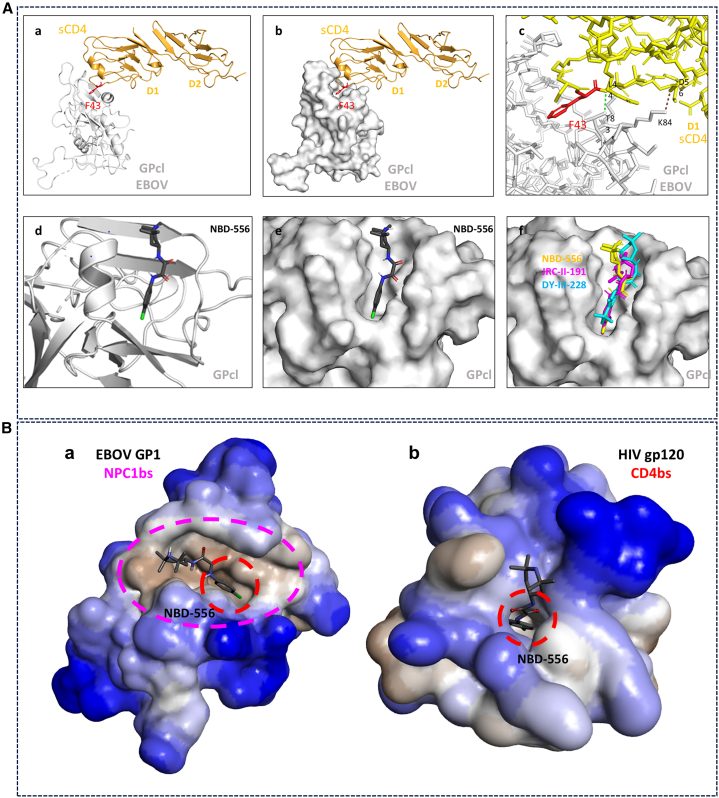
Molecular docking analysis of sCD4 and CD4mcs (A) Molecular docking analysis of sCD4 and CD4mcs binding to the EBOV-GP. sCD4 docking using HDOCK program. The docking energy is −150.41 kcal/mol. CD4mcs docking using AutoDock program. (a) sCD4-RBD ribbon model; (b) sCD4-RBD surface binding model; (c) sCD4-RBD interactions: hydrogen bond: *GP T83:OG1-CD4 L44:N (green)* and salt bridge: *GP K84:NZ-CD4 D56:OD2 (brown)*; (d) NBD-556 docking ribbon model (−6.477 kcal/mol); (e) NBD-556 docking surface model; and (f), superimposed of NBD-556 (yellow), JRC-II-191 (magenta), and DY-III-228 (cyan). (B) Comparisons of two receptor binding sites of CD4bs and NPC1-bs. (a) CD4bs^6^^,^^42^ of HIV gp120 (based on PDB 1G9N, YU2 strain). (b) NPC1bs^29^^,^^30^ of EBOV-GP (based on PDB 5F1B, Zaire EBOV). Showing the accommodation of CD4-mimetic compound NBD-556 in the red dash circle. Hydrophobic surface in gray; CD4bs of HIV gp120 in magenta dash circle; NPC1bs of EBOV-GP in red dash circle.

Molecular docking was utilized to test whether sCD4 and CD4mcs can dock to the NPC1 RBS. HDOCK,^38^^,^^39^ which is specific for protein-protein docking, was applied for sCD4/GP-RBD. The results demonstrate that sCD4(2D) can successfully dock to the RBS of EBOV-GP with a high free-energy of docking (−150.48 kcal/mol) in which one hydrogen bound and one salt bridge were observed (Figures 6Aa–6Ac). For CD4mcs docking, we used the AutoDock Vina program.^40^^,^^41^ The docking results also demonstrate that CD4mcs such as the prototype NBD-556 can dock at the RBS of EBOV-GP with a free-docking energy of −6.477 kcal/mol (Figures 6Ad and 6Ae). Two other analogs JRC-II-191 and DY-III-228 were also docked to this RBS (Figure 6Af). We notice that modification of region I by diverse groups such as the fluoro group, methyl group, and F-Cl-F groups affect the docking energies, which implies direct involvement of region I for the inhibition activity. The methyl groups of region III are also contacting the RBS of EBOV which is different from binding to HIV gp120 (see discussion).

## Discussion

Here, we report that sCD4, CD4-derived proteins, and some CD4mcs can inhibit EBOV infection (Table 1). Moreover, we have uncovered the mechanism of inhibition, suggesting that these CD4-related molecules target the GP binding site for the receptor NPC1 to block viral entry. The evidence includes sCD4 and CD4mcs binding to the RBD, while binding competition assays with the receptor NPC1 have confirmed the targeting site of GP-RBD. The mechanism is reminiscent of that used by sCD4 and CD4mcs to inhibit HIV infection. We can therefore conclude that there are similarities between the two ligands as well as the two binding sites (Figures 6A and 6B). Structurally, both CD4bs and NPC1bs have a hydrophobic deep cavity that is able to accommodate the phenyl ring of a protruding loop-based phenylalanine residue on both CD4 and NPC1 receptors.^6^^,^^29^^,^^30^^,^^43^ Specifically, HIV gp120 binds the CD4-binding loop-based Phe-43, while EBOV GP binds the NPC1 loop2-based Phe503.^6^^,^^29^^,^^30^^,^^43^ Similarly, this structural motif (phenyl ring) is also present on CD4mcs (such as NBD-556, JRC-II-191, and DY-III-228) and is inserted into the hydrophobic binding cavity. Modifications of the prototypical compound NBD-556 indicate that the phenyl ring of region I directly interacts with the NPC1 receptor-binding site (RBS) and blocks viral entry (Figures 6A, 6B, and S1). Molecular docking has demonstrated that sCD4 can dock to the NPC1 RBS of EBOV-GP. CD4mcs can also dock to this RBS, which supports their targeting of the RBS to interfere with NPC1 binding and reduce the viral entry. The region I phenyl ring of CD4mcs was found to be inserted into the deep cavity of RBS, resembling the phenyl ring of CD4-F43. This molecular similarity could explain why both sCD4 and CD4mcs could inhibit EBOV infection, since both can bind the NPC1 RBS. In addition, region III of CD4mcs is also important for the inhibition. Changes that increase or decrease the size of region III reduced or eliminated the inhibition. From the docking studies, region III appears to also make direct contact with the EBOV NPC1bs (Figures 6A and 6B). The hydrophobic methyl groups of region III may contribute to the hydrophobic interactions important for the binding. In addition, we have tested more than thirty CD4mcs and only a few exhibit the activity that implies the difference of EBOV NPC1bs from HIV CD4bs. Furthermore, we found that the more specifically designed compounds for HIV showed lower potency for EBOV, given that the two targets have some similarity but are not identical. Comparing their structures, it is obvious that CD4bs is deeper but smaller, NPC1bs is wider but shallower (Figure 6B). Futural structural studies will define these interactions better and provide a foundation for the improvement of antiviral activity.

**Table 1:** Summary of sCD4 and major CD4-mimetic compounds tested

Inhibitor	Cytotoxicity (CC_50_ μM)	EBOV (μM)	BDBV (μM)	Infectious EBOV (μM)
**sCD4s**				

sCD4 (2D)	–	4.46 ± 0.4	–	–
sCD4 (4D)	–	2.76 ± 0.6	1.74 ± 0.3	1.07
CD4-IgG	–	1.27 ± 0.7	–	–
CD4-IgG2	–	1.31 ± 0.2	0.75 ± 0.1	–

**CD4mcs**				

NBD-556	67.7 ± 7.3	18.21 ± 1.0	16.8 ± 2.1	18.78
NBD-557	–	19.76 ± 1.7	10.61 ± 1.8	–
JRC-II-191	43.18 ± 6.0	9.52 ± 1.4	9.99 ± 1.1	5.25
BNM-III-170	–	>30	–	–
TS-II-227	72.02 ± 7.8	25.73 ± 2.6	N/D	–
Compd N9	>100	9.51 ± 1.6	17.60 ± 2.7	9.46
DY-III-211	95.49 ± 6.1	21.26 ± 4.2	N/D	–
DY-III-226	48.42 ± 5.4	11.20 ± 0.8	10.64 ± 0.5	–
DY-III-227	68.28 ± 4.3	12.47 ± 1.5	12.14 ± 0.7	–
DY-III-228	20.55 ± 2.6	1.98 ± 0.5	2.25 ± 0.2	0.56

∗The validation of inhibition of IC_50_s by infectious virus was conducted in BSL-4 containment laboratory. Outcomes were similar to that seen with the pseudotyped virus platform. One example in more detail is included in Figure S5 for compound JRC-II-191 neutralizations against EBOV. This pseudotyped platform (pSGΔEnv and TZM-bl cells) was also verified by another pseudotyped platform (pNL4-3.Luc.R-E− and HeLa cells) (see more details for sCD4 neutralization in Figure S6). Concentration of cytotoxicity 50% (CC_50_); N/D, not determined. The mean values and standard deviation (mean ± SD) are calculated from three independent experiments.

Another interesting finding is that the sCD4 binding to NPC1bs shows quite different affinities between neutral pH and acidic conditions. This is reminiscent of the behavior of the NPC1 receptor, as EBOV entry occurs in late endosomes or lysosomes, where acidic conditions would be required to increase the receptor binding affinity.^44^ This finding implies that both sCD4 and NPC1 or their cognate binding partners may undergo significant conformational changes to increase binding affinity under acidic conditions.

In conclusion, our findings will open avenues for developing therapeutics to combat EBOV infection and other filoviruses such as Marburg virus (MARV), since MARV also uses NPC1 as the receptor for entry into the cells.^45^^,^^46^^,^^47^ The small-molecule CD4mcs will provide a novel class of inhibitors for drug development, these could be even more effective against EBOV than HIV, because sCD4 and CD4mcs are agonists for HIV infection, acting in *trans* to induce entry into cells that have low or negative CD4 levels.^12^^,^^14^ However, this should not apply to EBOV as it enters the cells with a mechanism that is independent of CD4 receptors. Although CD4 has an affinity with the EBOV comparable to that of its receptor NPC1, the difference is that CD4 molecules are present on human cell surfaces, but EBOV entry occurs exclusively either at endosomes or lysosomes. The way by which the EBOV RBD becomes exposed after protease cleavage in the endosomes may be involved in this distinction. Additionally, whether natural CD4 is involved in EBOV infection or pathogenesis in any way will need to be further explored.

### Limitations of the study

This study reported that human sCD4 protein can inhibit EBOV infection by targeting the receptor NPC1 binding site. Since CD4 is the primary HIV receptor, the CD4 and CD4-mimetic small molecules (CD4mcs) have been well studied in anti-HIV infection. Thus, we were able to CD4mcs to study the underlying molecular mechanism of sCD4 inhibition of EBOV. The validation of the mechanism requires the resolution of binding structures of sCD4 with the receptor-binding domain, which will be needed for further studies.

## Resource availability

### Lead contact

Further information and requests for resources and reagents should be directed to and will be fulfilled by the lead contact, Shi-Hua Xiang (sxiang2@unl.edu).

### Materials availability

All materials generated in this study will be made available on request.

### Data and code availability

All data are available in the main text or the supplemental information. This paper does not report the original code. Any additional information is available from the lead contact upon request.

## Acknowledgments

We thank Dr. Joseph Sodroski (Harvard Medical School) for critical review of the manuscript. Christopher Fritschi (Uppen) for sending CD4-mimnetic compounds, and the UNL Microscopy core facility (You Zhou and Bera Altartouri) for helping with imaging of GP-RBD transected cells. The following reagents were obtained through the HIV Reagent Program, Division of AIDS, NIAID, NIH: human soluble CD4-183 (sCD4-183) two-domain protein, recombinant from *Escherichia coli*, ARP-7356, contributed by Pharmacia, Inc.; human soluble CD4 (sCD4, 4D) protein, recombinant from CHO cells, and ARP-4615 and CD4-IgG2 fusion protein, ARP-11780, contributed by Progenics Pharmaceuticals, Inc.; human CD4-Ig recombinant protein, ARP-13058, contributed by Dr. Xueling Wu.; and Cf2Th synCCR5^+^ Cells, ARP-4662, contributed by Drs. Tajib Mirzabekov and Joseph Sodroski.

## Author contributions

S.-H.X. initiated and managed the project, and conducted protein sample evaluation and molecular docking. L.L.W. conducted all pseudovirus-based neutralization experiments as well as cell surface binding assay. D.A. and L.L.W. conducted flow cytometry analyses. J.S. performed sCD4 and NBD-556 binding assays using BLI method. P.K., J.J.P., B.E., M.R.H., and R.A.D. conducted all neutralization assays against infectious EBOV in the BSL-4 containment. D.Y. and A.B.S. provided most of CD4mc compounds including newly synthesized three compounds against EBOV. Y.L. and S.-H.X. conducted molecule docking.

## Declaration of interests

S.-H.X. and L.L.W. are inventors on US patent application no. 63/597563, on November 9, 2023, entitled “Methods and Compositions for Inhibiting Viruses”.

## STAR★Methods

### Key resources table

**Table:** 

REAGENT or RESOURCE	SOURCE	IDENTIFIER
**Antibodies**		

Alexa Fluor® 488 AffiniPure™ Rabbit Anti-His Tag	Jackson ImmunoResearch Lab	Code: 300-545-240
Alexa Fluor® 488 Anti-Mouse IgG (H+L)	Invitrogen	-
EBOV, GP2 mouse Mab	Sino Biological Inc.	40442-MM28

**Bacterial and virus strains**		

Ebola Zaire virus	NEIDL	Mayinga C07
Ebola Zaire virus	NIH-IRF	Makona IRF0192

**Chemicals, peptides, and recombinant proteins**		

sCD4-183	NIH HIV Reagent Program	ARP-7356
Human Soluble CD4 (4D)	NIH HIV Reagent Program	ARP-4615
CD4-IgG2 Fusion Protein	NIH HIV Reagent Program	ARP-11780
CD4-Ig Recombinant protein	NIH HIV Reagent Program	ARP-13058
RBD-Fc (BDBV)	Sino Biological Inc.	40368-V02H1
NPC1-His	Sino Biological Inc.	16499-H32H
NBD556	Tocris Bioscience	CAT.NO.5811
NBD557	MedChemExpress	HY-76649
N9	ChemBridge	ID#7025333

**Experimental models: Cell lines**		

HEK293T	ATCC	CRL-3216
Vero-E6	ATCC	CRL-1586
HeLa,	NIH AIDS Reagent Program	ARP-153
TZM-bl	NIH AIDS Reagent Program	ARP-8129
Cf2Th-CCR5	NIH AIDS Reagent Program	ARP-4662

**Recombinant DNA**		

HIV-1 pSG3ΔEnv plasmid	NIH AIDS Reagent Program	ARP-11051
pNL4-3.Luc.R-E- plasmid	NIH AIDS Reagent Program	HRP-3418
Zaire ebolavirus envelope glycoprotein genes	GenBank	AIO11753.1
EBOV GP-pcDNA3.1(+)	GenScript	Order ID 520191
Bundibugyo ebolavirus envelope glycoprotein genes	GenBank	AGL73460
BDBV GP-pcDNA3.1(+)	GenScript	Order ID U4274UMVG0
pEBOV-GPdeltacm plasmid	GenScript	Order ID U9172HK030
Plasmid pHEF Expressing Vesicular Stomatitis Virus (VSV-G)	NIH AIDS Reagent Program	ARP-4693
Amphotropic murine leukemia virus (A-MLV)	NIH AIDS Reagent Program	HRP-1065

**Software and algorithms**		

GraphPad Prism versions 10.0.3	GraphPad	https://www.graphpad.com/scientific-software/prism/

### Experimental model and study participant details

#### Virus strains

Ebola Zaire virus (Mayinga, or Makona C07(IRF0192). The viral envelope glycoprotein genes used for pseudotyped viruses were the Ebola virus (*Zaire ebolavirus*, GenBank: AIO11753.1), Bundibugyo ebolavirus (GenBank: AGL73460, Democratic Republic of the Congo) and the VSV-G was from Vesicular stomatitis virus (VSV) and A-MLV-GP from Amphotropic murine leukemia virus (A-MLV). These two systems were compared in Figure S6.

#### Cells

HEK293T (human embryonic kidney; ATCC CRL-3216), Vero-E6 cells (ATCC, 1586), HeLa, Huh7 cells and TZM-bl, Cf2Th-CCR5 cells (NIH AIDS Reagent Program, Division of AIDS, NIAID, NIH) were grown at 37°C in Dulbecco’s modified Eagle’s medium (DMEM) with 10% fetal bovine serum (FBS). All cell lines were confirmed for lack of mycoplasma contamination.

#### Proteins

sCD4-183 (ARP-7356), Two-Domain Protein, Recombinant from *Escherichia coli*, Human Soluble CD4 (4D) (ARP-4615) Protein, Recombinant from CHO Cells and CD4-IgG2 Fusion Protein (ARP-11780 or PRO 542) either obtained from NIH HIV Reagent Program or purchased from Lantheus Medical Imaging. Human soluble CD4 (4D) protein (CD4-3167H) was also purchased from Creative Biomart Inc. CD4-Ig Recombinant protein was obtained from NIH HIV Reagent Program. RBD-Fc (BDBV), NPC1-His proteins were purchased from Sino Biological Inc.

#### Small compounds

NBD-556 was purchased from Tocris Bioscience and NBD-557 from MedChemExpress (MCE). Compound N9 (ID#7025333) was purchased from ChemBridge. Compounds JRC-II-191, BNM-III-170, DY-III-226, DY-III-227, DY-III-228, and other CD4-mimetic compounds tested were provided by Dr. Amos Smith III (Table S1).

### Method details

#### Pseudotyping viruses

All pseudotyped viruses are based on HIV-1 backbone. Two backbone plasmids were used: HIV-1 pSG3ΔEnv and HIV-1 pNL4-3.Luc.R-E- (NIH AIDS Reagent Program). HIV-1 backbone plasmid pSG3ΔEnv or pNL4-3.Luc.R-E- was used for making pseudotyped viruses. The glycoprotein genes (GPs) of Ebola viruses (Zaire ebolavirus, EBOV and Bundibugyo ebolavirus, BDBV) were synthesized and cloned into the pCDNA3.1(+) expression vector. Both plasmids of pSG3ΔEnv (2μg) and the GP envelope (6μg) were co-transfected into 293T cells in a 10 cm plate using 24 μg of transfection reagent polyethyleneimine (PEI). The plates were cultured in a tissue incubator at 37°C and 5% CO_2_ for two days, then the medium was harvested and centrifuged at 5000 rpm for 10 min to remove cell debris. The supernatants containing the pseudotyped viruses were made aliquots and stored at -80°C.^48^

#### Inhibition assay against pseudotyped Ebola viruses

Inhibition assay was performed in a 96-well plate using pseudotyped viruses and TZM-bl cells (6000/well) as this cell-line has a Luciferase reporter gene under the inducible promoter of Tat protein factor. The mixtures of viruses and compound samples were transferred onto the target cell wells for infection. One-day post infection, the media were removed, the cells were washed once with PBS and incubated in fresh media for one more day. Then the cells were lysed in 1x Passive Lysis Buffer (Promega) and kept at room temperature for 20 minutes. The luciferase activity was measured using luciferin substrate (Promega) in a Veritas Luminometer. All the samples tested in triplicates and the neutralization activities were calculated in comparison with controls of positive (virus only) and negative (cells only).^49^

#### Inhibition assay against replication-competent Ebola^50^^,^^51^

The inhibition assay against wild-type infectious Ebola virus was conducted by Dr. Robert Davey lab in Boston University and Dr. Michael Holbrook in NIH Integrated Research Facility at Fort Detrick. Their methods were described previously.^48^^,^^51^^,^^52^

##### Virus and cell preparation

Vero E6 cells (ATCC) were maintained in Dulbecco Modified Eagle Medium (DMEM; Gibco, 11995073, Gaithersburg, MD, USA) supplemented with 10% Heat Inactivated Fetal Bovine Serum (Gibco, 10500064) (DMEM-10) at in a humidified incubator at 37°C and 5% CO_2_ overnight. For Neutral Red Agarose Overlay (NRAO) plaque assays, cells were plated on 6-well tissue culture plates and taken into the BSL-4 at 90–100% confluency. For crystal violet plaque assays, cells were protected with 1% Penicillin/Streptomycin L-Glutamine (Lonza, 17-718R, Basel, Switzerland) in their DMEM-10 medium, plated on 6-well tissue culture plates, and taken into the BSL-4 at 75–90% confluency. TCID_50_ (Median Tissue Culture Infectious Dose) assays were plated on 96 well tissue culture plates in DMEM supplemented with 2% FBS (DMEM-2) and 1% Penicillin/Streptomycin L-Glutamine (Lonza, 17-718R) allowed to grow overnight before entry into the BSL-4. After entry into the BSL4, plates were inoculated with Ebola virus Zaire Mayinga (EBOV). Focus-Forming Assay cells were plated on either 96 (180 μL in the first row, 100 μL in the remainder) or 384 well (25 μL in each well) plates with DMEM-10 and taken into the BSL4 at 75–90% confluency after overnight incubation. Plates were inoculated with EBOV.

##### Overlay preparation

50 mL of NRAO primary overlay consisted of 25 mL Minimal Essential Medium Eagle with Earle’s BSS (EMEM, Lonza, 12-668E) buffered with 4% FBS, 2% L-Glutamine (Lonza, 17-905C), and 2% Sodium Pyruvate (Lonza, 13-115E) with 25 mL Agarose (1:1 mixture). 50 mL NRAO secondary overlay consisted of 25 mL EMEM buffered with 4% FBS, 2% L-Glutamine, 2% Sodium Pyruvate, and 8% Neutral Red (Gibco, special formulation) with 25 mL Agarose (1:1 mixture). Methylcellulose overlay consisted of 500 mL DMEM-2 with 1% Penicillin/Streptomycin L-Glutamine and 100 mL Methylcellulose (16.67%) (Sigma, M0387, St. Louis, MO, USA) All overlays were heated to at least 37°C before adding.

##### NRAO assay infection with filovirus

Once inside the BSL-4, the virus was retrieved, and a virus dilution series was prepared. 100 μL of virus was added to 900 μL DMEM-2, creating a ten-fold dilution. Virus was then serially diluted from 10^−1^ to 10^−6^ by transferring 100 μL virus/DMEM-2 across 6 tubes. In general, 0.5 MOI was used for virus neutralization assay. The samples were mixed well using a new pipette between each dilution step. The DMEM-10 in the 6-well plates was then decanted into 5% Microchem solution. 400 μL of each dilution was plated in each well, beginning with the 10^−2^ to the 10^−6^ dilution. Adding 400 μL per well allowed for each dilution to be plated in duplicate. In the final well, 400 μL of DMEM-2 was added as an uninfected control. After the virus was administered, plates were placed on a rocking platform within a humidified incubator at 37°C, 5% CO_2_ for 1 h.

After 1 h incubation, plates were removed from the incubator and inoculum was removed from each well by pipette and disposed of in 5% Microchem. Primary overlay was added at 2 mL in a drop-wise fashion to each well. The agarose overlay was allowed to solidify before incubating for 7 days at 37°C, 5% CO_2_. After 7 days of incubation, 2 mL secondary overlay was added in a drop-wise fashion. Plates were placed back into the incubator overnight. The next day, plates were scanned using a flatbed scanner and scan files were assessed for viral titer. Wells with 15–150 plaques were considered within acceptable limits. The viral titer was calculated based on the number of plaques multiplied by the dilution factor and the correction factor of 2.5 (for 1 mL) and is reported in pfu/mL.

##### CVMO assay^51^

Once inside the BSL-4, virus was retrieved, and a virus dilution series was prepared. 100 μL of virus was added to 900 μL PBS, creating a ten-fold dilution. Virus was then serially diluted further from 10^−1^ to 10^−6^ by transferring 100 μL virus/PBS down 6 tubes. Samples were mixed well using a pipette between each dilution step. The DMEM-10 in the 6-well plates was then decanted into 5% Microchem solution. 400 μL of each dilution was plated in each well, beginning with the 10^−2^ to the 10^−6^ dilution. Adding 400 μL per well allowed for each dilution to be plated in duplicate. In the final well, 400 μL of PBS was added as an uninfected control. After the virus was added, plates were placed on a rocking platform within a humidified incubator at 37°C, 5% CO_2_ for 1 h.

After 1 h incubation, plates were removed from the incubator and inoculum was removed from each well by pipette and disposed of in 5% Microchem. Methylcellulose overlay was added at 5 mL in a drop-wise fashion to each well. The assay was then incubated for 10 days at 37°C, 5% CO_2_. After 10 days of incubation, methylcellulose overlay was removed from each well, plates were submerged in 10% formalin for inactivation and incubated overnight at 4°C. After inactivation, plates were removed from the BSL-4 and washed with 1x PBS. Plates were next stained with 2 mL CVMO for 10 min at room temperature before washing excess staining material from the plate. Plaques were counted using a light microscope, with 25–250 plaques considered acceptable limits. Viral titer was calculated based on the number of plaques multiplied by the dilution factor and the correction factor of 2.5 (for 1 mL) and is reported in pfu/mL.

##### TCID_50_ assay

Once inside the BSL-4, media was removed from the wells by decanting into 5% Microchem and washed with 200 mL PBS. The PBS wash was discarded and 180 μL DMEM-2/antibiotics was added to each well, with 20 μL virus placed into all wells of the first column. A 10-fold serial dilution series was completed by moving 20 μL increments down each row of the plate. Plates were then incubated for 10 days at 37°C, 5% CO_2_.

After incubation, plates were decanted into 5% Microchem. 100 μL CVMO stain was added to each well, and allowed to incubate at room temperature for 10 min. Plates were next decanted and thoroughly washed with water to remove excess staining material. Once dried, plates were assessed for cytopathic effect (CPE) in each column. The final titer was calculated using the Reed–Muench Method.^51^

##### Focus forming unit (FFU) assay

Inside BSL-4 containment, 20 μL (96 well) or 25 μL (384 well) of virus was added to each well of the first column. A half serial dilution series was completed by 100 μL (96 well) or 25 μL (384 well) increments down each row of the plate. Plates were then incubated 36–48 h in a humidified incubator at 37°C, 5% CO_2_. After incubation, plates were decanted into 5% Microchem. Plates of both well types were submerged in 10% formalin for inactivation and incubated at 4°C overnight. Plates were then removed from the BSL-4 and stained with antibody in 3.5% BSA and Hoechst 33,342. The concentration for Ebola Zaire primary antibody was 1:1500 with a mouse mAb anti-GP (IBT, 0201-020, Rockville, MD, USA). For Sudan virus, a concentration of 1:1000 was used in a mouse mAb anti-GP (IBT, 0280-001). Cell nuclei were stained with Hoechst 33,342 at a 1:10,000 concentration. Plates were next imaged and then analyzed for virus titer using CellProfiler.

#### Cell viability assay (MTT)

Cell viability was measured by MTT assay. The TZM-bl cells (3000/well) were seeded in a 96-well plate and incubated for 24 h at 37°C. Media was removed and replaced with 100 μl of compound solution in triplicates for two days and then replaced the peptide solution with 100 μl complete DMEM for continuingly culturing for one more day. The cultured media were removed, and the cells were washed once with PBS for analysis. A 50 μl solution of 5 mg/ml MTT [3-4,5-dimethylthiazol-2-yl)-2,5-diphenyltetrazolium bromide] (Sigma-Aldrich, MO, USA) was added to each well. The plates were incubated for 3 h at 37°C, and the absorbance was measured at 570 nm and with 650 nm (background) wavelength.

#### Flow cytometry

Cells (293T or Cf2thCCR5) were transfected with pEBOV-GPdeltacm plasmid and quantified for RBD surface expression using anti-RBD antibody (Polyclonal antibody anti-EBOV-GP-RBD, Invitrogen). The transfected cells were used for binding competition analysis. In general, 20 μM of NPC1-his used and quantified for about 90% positions reached and the competition was conducted by adding different concentrations of sCD4 (20 μM) or NBD-556 of 10 μM, 20 μM and 40 μM. After ligand binding 45 min at room temperature, the cells were washed and stained with 0.01 ug/ul of anti-his antibody for 30 min, then the cells were washed twice with PBS and resuspended in 100ul PBS.

The stained Cells were resuspended in 100 μL PBS and analyzed on a Beckman Coultier CytoFlex LX using 488 nm and 594 nm wavelengths. Flow-Jo software was used for generating final plots. Stained wild-type cells and sample cells with only secondary antibody. The data was acquired on a Beckman Coulter CytoFLEX LX system. The Alexafluor 488 signal was excited by a 488nm blue laser, and the emission was collected using a 525/40 nm band pass filter. Gating for analysis is determined based on both unstained cells and stained cells with the secondary marker only. At least 10K events were collected and analyzed. The antibody only stained was used as controls to determine background fluorescence and set appropriate gates.

#### Confocal microscopy

Cells were harvested and washed with PBS and resuspended in a volume of 100 uL 4% paraformaldehyde (PFA) for fixation. After one hour, cells were washed with PBS to remove PFA and resuspended 100 μL PBS. Next, 1 μL of primary antibody specific to His-tag was added, and the mixture was given one hour with occasional mixing for antibody interaction. Afterwards, the cells were pelleted, washed to remove unbound antibodies, and resuspended in 100 μL PBS. 1 μL of secondary antibody conjugated to AlexaFluor 488 was added, and the mixture was again allowed to interact for one hour. Following this, the cells were again washed to remove unbound antibody and then analyzed via either confocal microscopy or flow cytometry. For confocal microscopy, the cells were pelleted, resuspended in a volume of 20 μL PBS, and placed on a microscope slide with coverslip. They were then analyzed on a Nikon A1R-Ti2 confocal microscope, and images were captured using Nikon NIS-Elements Imaging software.

#### Bio-Layer Interferometry (BLI) analysis

For 2D-CD4 binding, samples of fusion receptor, GP-RBD-FC, were diluted 50-fold to 2 μM into either PBS pH 7.4 or 0.1M MES pH 6.1 buffers. A total of 4 μL were used to load the AHC biosensors (Sartorius, Gottingen, Germany) which were preloaded with the Anti-hIgG Fc capture antibodies. The samples of the 2D-CD4 receptor were desalted into the same buffers using Pall (Cytiva) Nanosep centrifugal filters (Cytiva-Danaher, Washington, DC) to a final concentration of 200 μM, as determined by the UV-visible spectrum. The experiments were run as indicated by the Sartorius Octet N1 software, with five steps for equilibration (30 sec.), loading RBD (60 sec), washing (60 sec), ligand binding (60 sec.) and dissociation (120 sec). The same buffers, either pH 6.1 or 7.4, were used for equilibration, washing and dissociation. The raw data was analyzed with the software for the Octet N1 (Bio-blitz) and plotted using Sigmaplot 14 (Grafiti, Palo Alto, CA).

For NBD-556 binding, a stock of GP-RBD-FC was prepared at 2 μM into either PBS pH 7.4 or 0.1M MES pH 6.1 buffers, which was loaded onto the anti-human lgG Fc antibody BLI chips. After the baseline, receptor loading steps, the BLI sensor was equilibrated with a solution of the NBD-556 in the corresponding buffer at the desired concentration. This was followed by binding of 50 μM 2D-CD4 in the same buffer and with the same NBD-556 concentrations, followed by a final dissociation step using the equilibration buffer. The experiments were run as indicated by the Sartorius Octet N1 software, with five steps for equilibration (30 sec.), loading RBD (60 sec), washing (60 sec), ligand binding (60 sec.) and dissociation (120 sec). The same buffers, either pH 6.1 or 7.40, were used for equilibration, washing and dissociation. The amplitudes of CD4 binding were plotted against the NBD-556 concentrations using Sigmaplot 14 (Grafiti LLC) and fitted with non-linear regression to a single binding hyperbola. We observed no effect of the presence of the NBD-556 on the binding and dissociation rates of CD4 to the RBD receptor.

#### Molecular docking

Soluble CD4 (2D-CD4, from PDB 1G9N) was docked to EBOV-GP-RBD (GPcl, from PDB 5F1B) using HDOCK program^38^^,^^39^ for protein-protein interactions. The input specific residues of GP were V79, T83, W86, F88, L111, V141 and P146). Dockings for CD4-mimetic small molecules (CD4mcs) were conducted using AutoDock Vina.^40^^,^^41^ All structural analysis was performed using Discovery Studio Visualizer (BIOVIA), Chimera (UCSF) and PyMol (Schrödinger, Inc).

#### Compound synthesis

##### General procedures

All reactions were conducted in oven-dried glassware under an inert atmosphere of nitrogen, unless otherwise stated. All solvents were reagent or high-performance liquid chromatography (HPLC) grade. Anhydrous dichloromethane (DCM) was obtained from the pure solveTM PS-400 system under an argon atmosphere. All reagents were purchased from commercially available sources and used as received. Reactions were magnetically stirred under a nitrogen atmosphere, unless otherwise noted, and were monitored by thin layer chromatography (TLC) which was performed on pre-coated silica gel 60 F-254 plates (40-55 micron, 230-400 mesh) and visualized by UV light or staining with KMnO_4_ and heating. Yields refer to chromatographically and spectroscopically pure compounds. Proton (^1^H) and carbon (^13^C) NMR spectra were recorded on a Bruker Avance III 500-MHz spectrometer. Chemical shifts (δ) are reported in parts per million (ppm) relative to chloroform (δ 7.26) for ^1^H NMR, and chloroform (δ 77.2). High resolution mass spectra (HRMS) were recorded at the University of Pennsylvania Mass Spectrometry Service Center on either a VG Micromass 70/70H or VG ZAB-E spectrometer. Lyophilization was performed in a Labconco FreeZome 12 Plus lyophilizier (0.148 mb ). The purity of new compounds was judged by NMR and LCMS (>97%). (see detailed procedures in the supplemental information: Data S1).

### Quantification and statistical analysis

GraphPad Prism version 10.0.3 for Windows (San Diego, CA, USA) was used for statistical analysis of virus neutralization and cytotoxicity data. The virus neutralization data were analyzed by fitting the percentage of virus inhibition at different compound (proteins or small molecules) concentrations to a non-linear regression curve, using the regression model. The data were plotted as percent neutralization versus log_10_-transformed compound concentration. The IC_50_ values, representing the concentration at which 50% virus neutralization was achieved, were calculated from the fitted curves. Goodness of fit was assessed by R^2^ values which was also shown in each neutralization curve. The *P*-values < 0.05 were considered statistically significant. The CC_50_ values (cytotoxic concentration 50%) were also calculated from the normalized absorbance measured by MTT assay into percentage values and then fit a non-linear regression curve to get the CC_50_ values using the GraphPad Prism software.
