# Supplementary material for: Soluble CD4 inhibits Ebola virus infection by targeting endosomal receptor-binding site
Source: iScience. 2025 May 2;28(6):112573. doi: 10.1016/j.isci.2025.112573 (PMC12148371; doi:10.1016/j.isci.2025.112573)
Supplement: Document S1. Figures S1–S7, Table S1, and Data S1 [file mmc1.pdf]

iScience, Volume *28*

Supplemental information

Soluble CD4 inhibits Ebola virus infection

by targeting endosomal receptor-binding site

Leah Liu Wang, Patrick Keiser, Derek Yang, Javier Seravalli, J.J. Patten, Brett Eaton, Dirk Anderson, Yi Liu, Michael R. Holbrook, Amos B. Smith III, Robert A. Davey, and Shi-Hua Xiang

Fig. S1

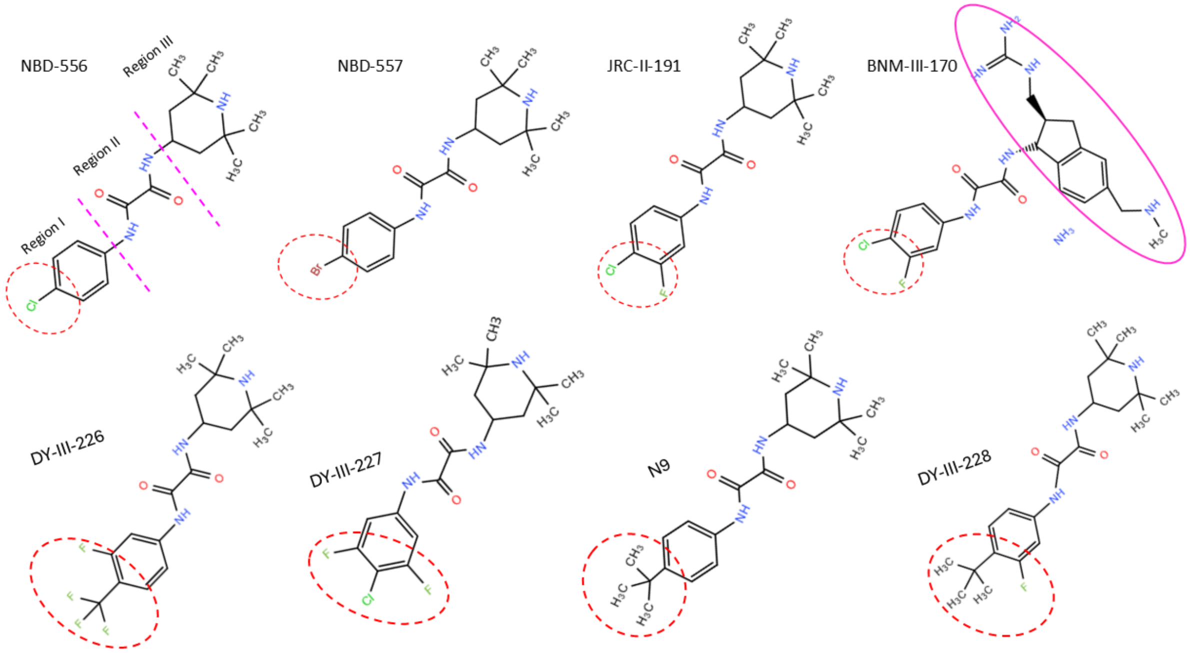

Fig. S1. Structural comparisons of CD4-mimetic small molecules (CD4mcs). The prototypical compound NBD-556 consists of three regions: region I (substituted phenyl ring), region II (oxalamide linker), and region III (tetramethyl piperidine). All region I modifications are circled in red dash line. The large group of region III in compound BNM-III-170 is circuited in magenta.

Fig. S2

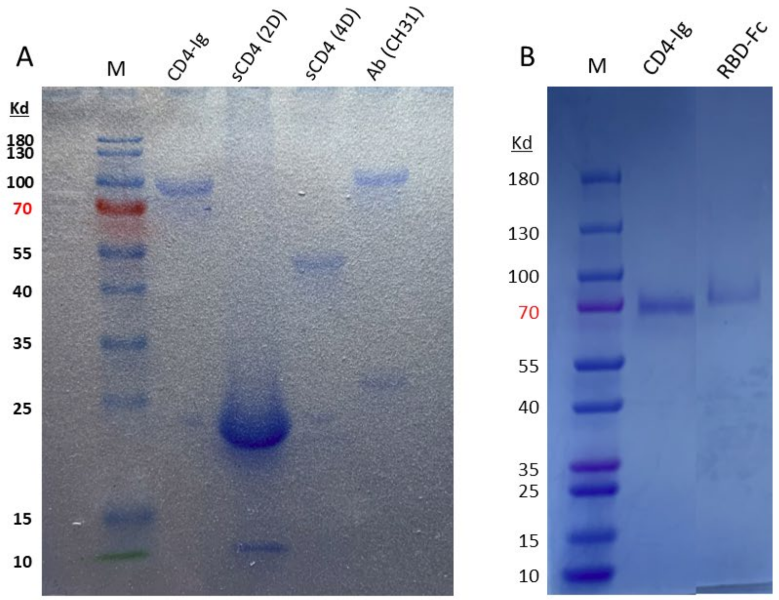

Fig. S2. Protein sample analysis of sCD4 and NPC1-RBD in the PAGE gels. The protein-based samples are analyzed in the PAGE gel for the size and purity. A. Marker (M) sCD4 (2D), sCD4 (4D), CD4-Ig, RBD-Fc (BDBV), Antibody control VRC-CH31 (ARP-12565, NIH AIDS program). B. Marker (M), CD4-Ig and RBD-Fc.

Fig. S3

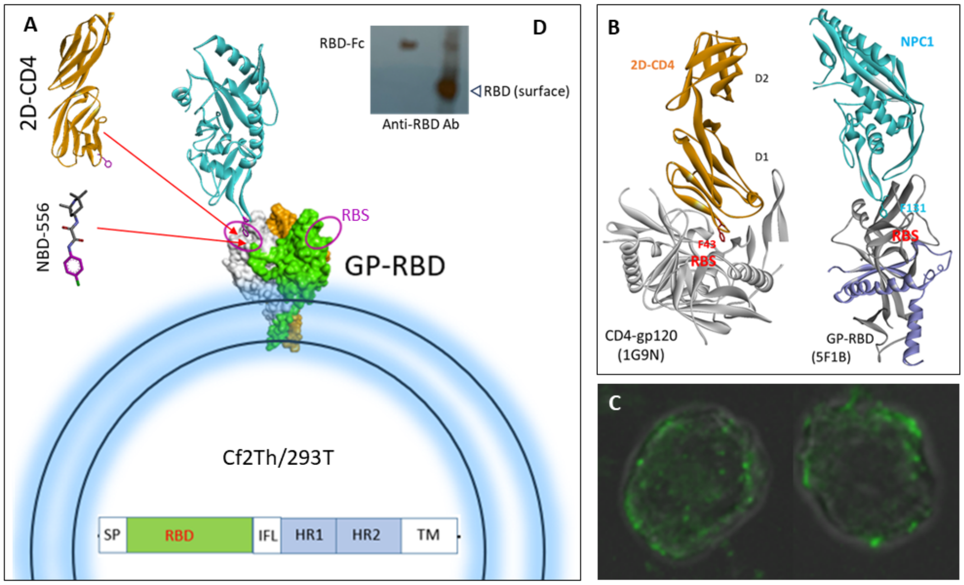

Fig. S3. Cell surface binding competition method designed. A. Bundibugyo ebolavirus (BDBV) GP-RBD is displayed on the Cf2Th cells (Cf2ThsynCCR5$^+$) cell surface by transfecting the construct pEBOV-GPΔmc. **B**. Comparison of HIV gp120-CD4 (PDB 1G9N) [47] and EBOV-GP-NPC1-C (PDB 5F1B) [30]. **C**. Alexa Fluor 488 stained EBOV GP-RBD on the surface of 293T cells. **D**. EBOV GP-RBD recognized by EBOV-GP specific monoclonal antibody (EBOV GP-RBD Polyclonal, Zaier EBOV Mayinga 1976, Invitrogen).

Fig. S4

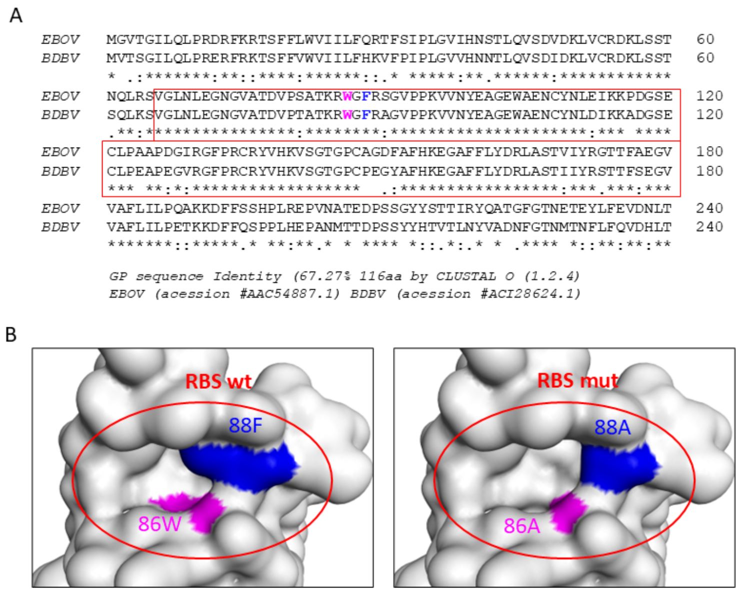

Fig. S4. Mutation models of WF/AA (86W/A and 88F/A) in RBD. (A). Protein sequence comparison of receptor binding domains (RBDs) of EBOV strains (Zaire EBOV and Bundibugyo ebolavirus, BDBV). **(B).** Surface models of WF/AA mutant. 86W/A in red, 88F/A in blue.

Fig. S5

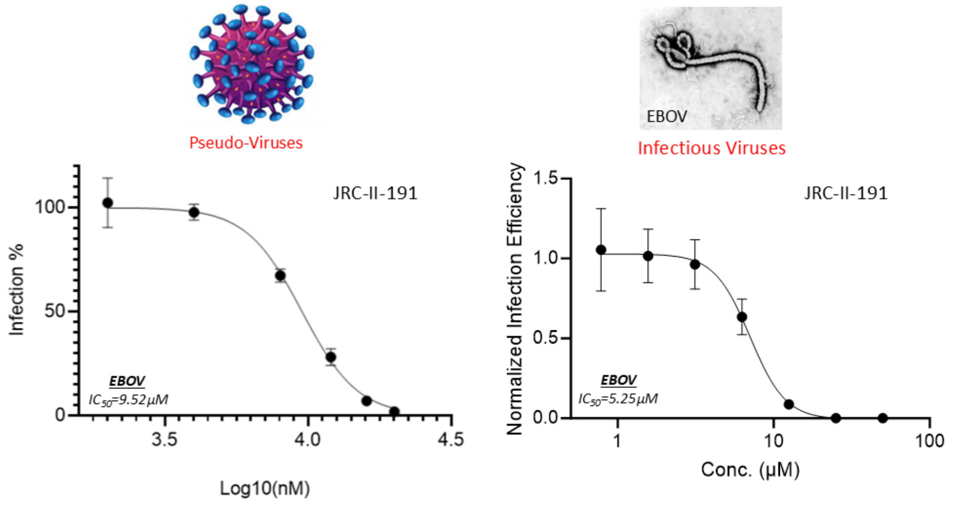

Fig. S5. Comparisons of the neutralization sensitivities of pseudotyped and infectious Ebola virus (EBOV). The evaluations of pseudotyped and infectious

platforms from the compound JRC-II-191 inhibitions by which the inhibition data are comparable.

Fig. S6

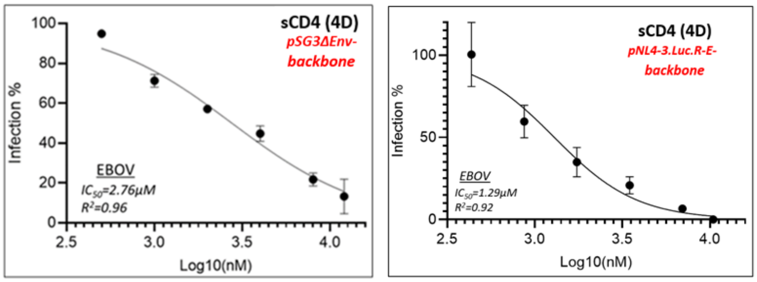

Fig. S6. Comparisons of two HIV backbones (pSG3ΔEnv and pNL4-3LucR-E-) based pseudotyped Ebola viruses. pSG3ΔEnv based psudotyped viruses were assayed in TZM-bl cells (from HeLa cell line that expresses CD4, CXCR4, and CCR5 and also, luciferase and β-galactosidase under the control of the HIV-1 promoter), but pNL4-3LucR-E- (defective Nef, Env and Vpr genes, but a firefly luciferase gene was inserted into the nef gene) based pseudotyped viruses were assayed in Hela cells. For example, the sCD4 (4D) inhibition data from both pseudotyped and wild-type viruses are comparable.

Fig. S7

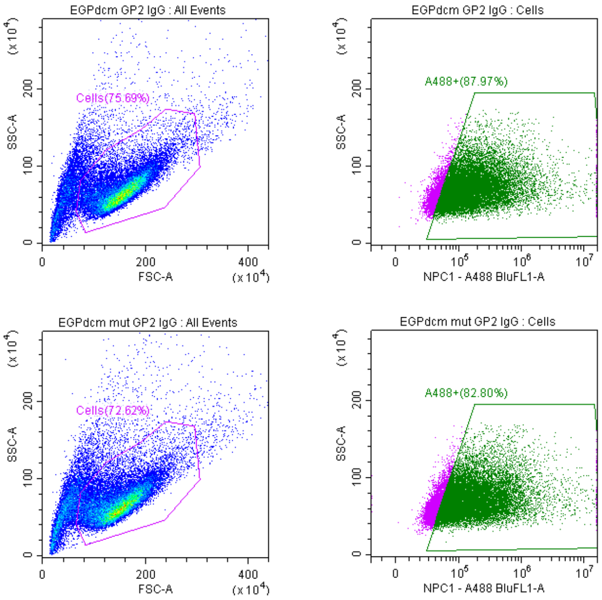

Fig. S7. Surface expression comparison of RBD mutant (EGPdcm mut, WF/AA) with its Wild type (EGPdcm) in the cell surface model. Specific anti-GP2 antibody (EBOV GP2 mouse Mab, Sino Biological Inc) and Alexa Fluor-488 Anti-Mouse IgG (Invitrogen) were used for Flow cytometry assay.

Tabel S1 CD4-mimetic compounds Tested

No	ID	mol MW	structure
1	NBD556	337.8	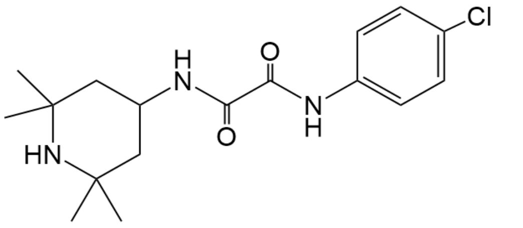
2	NBD557	382.3	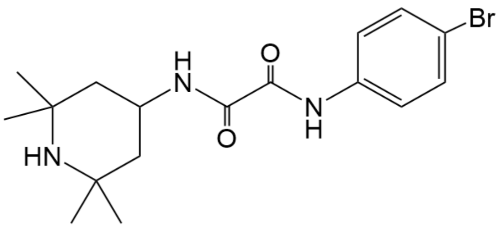
3	JRC-II-191	355.8	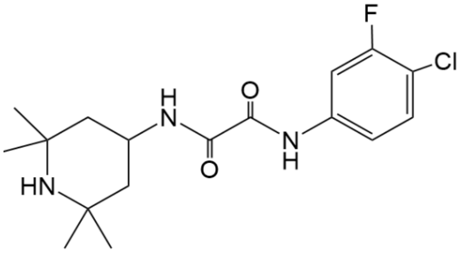
4	DY-III-226	389.4	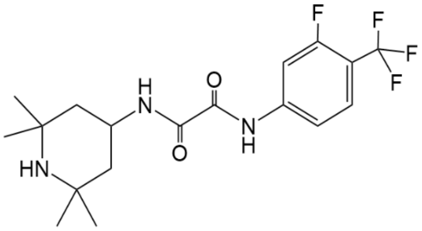
5	DY-III-227	373.8	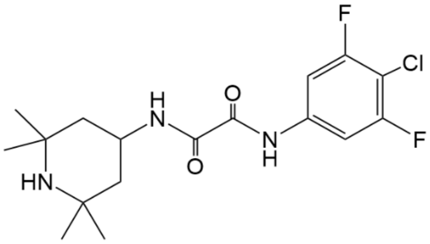
6	DY-III-228	377.5	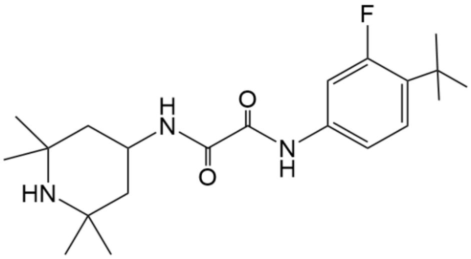

7	N9	359.5	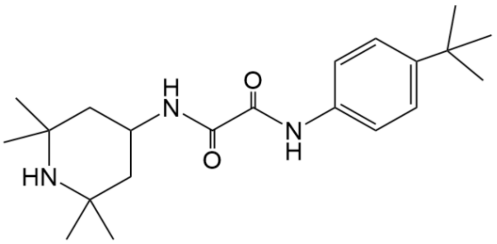
8	AS-II-207	313.8	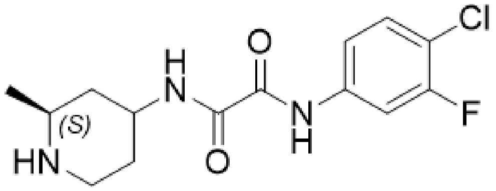
9	AS-II-195	313.8	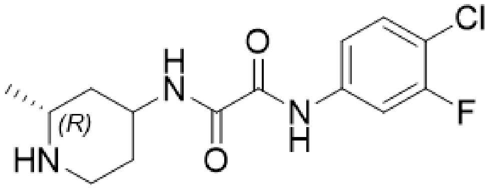
10	AS-II-144	360.8	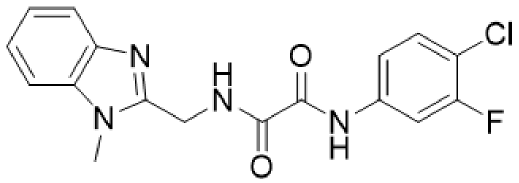
11	AS-I-229	433.9	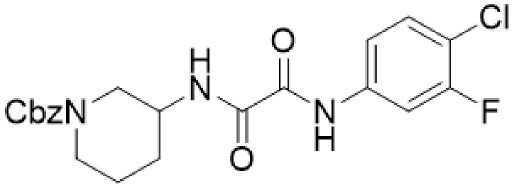
12	AWS-I-025	299.7	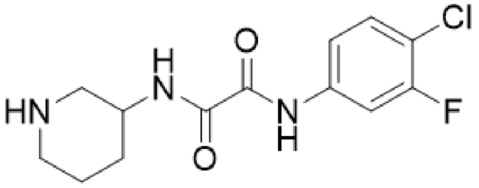

13	AS-II-143	368.7	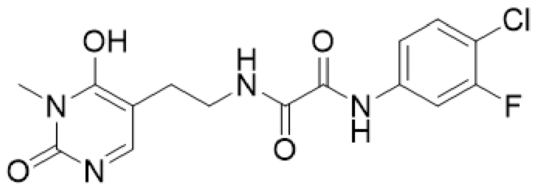
14	AWS-I-022	341.8	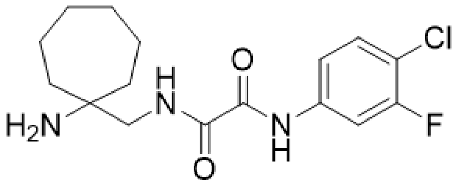
15	TS-II-229	341.8	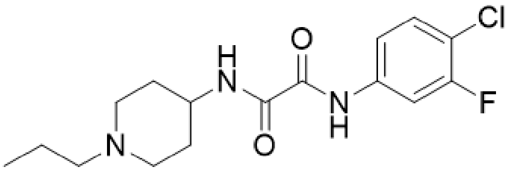
16	TS-II-228	327.8	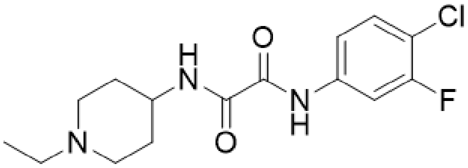
17	TS-II-227	313.8	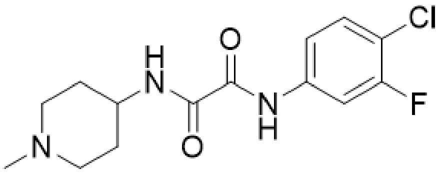
18	TS-II-196	299.7	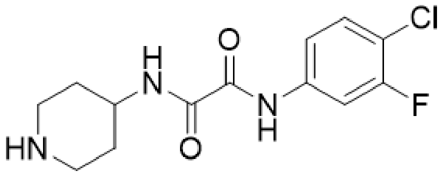

19	TS-II-197	284.7	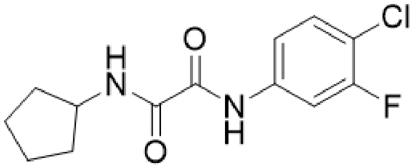
20	AS-II-242	367.8	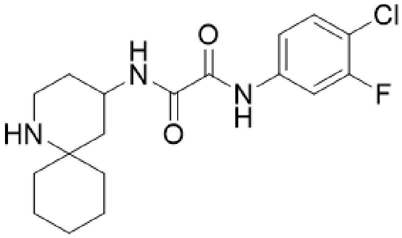
21	AS-II-246	405.9	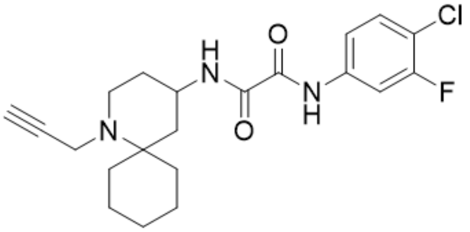
22	TS-I-150	371.8	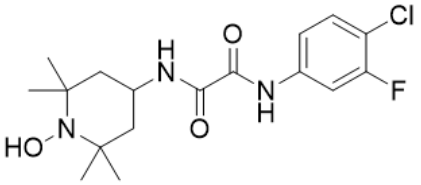
23	TS-II-211	353.8	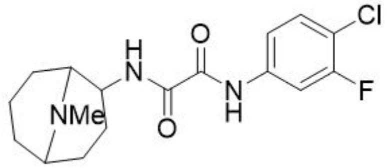
24	TS-II-226	341.8	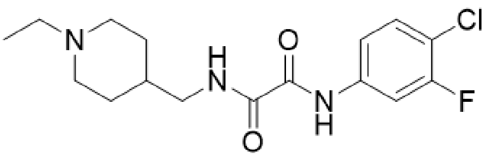

25	TS-II-225	327.8	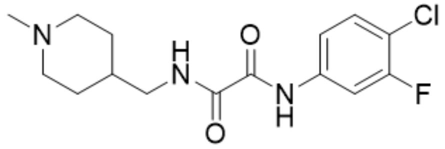
26	TS-II-198A	313.8	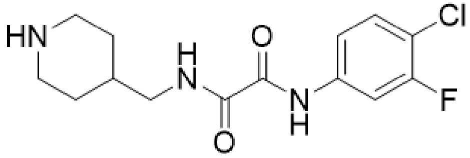
27	TS-II-068	465.3	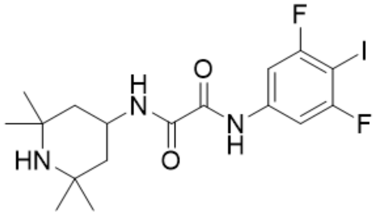
28	TS-I-162	382.8	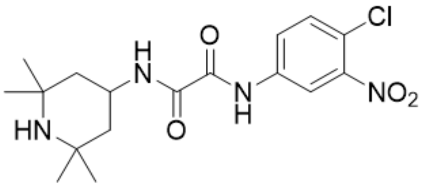
29	TS-II-055	513.6	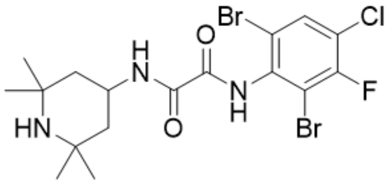
30	TS-I-204	353.8	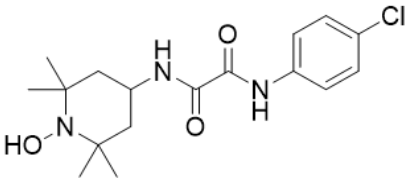

31	TS-II-230	481.3	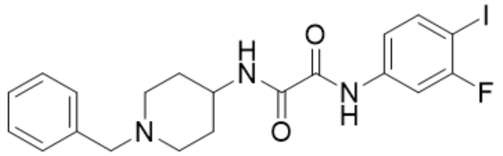
32	TS-I-187	410	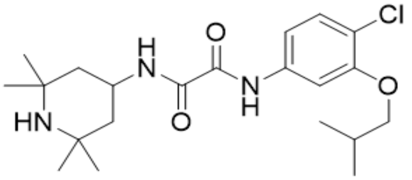
33	TS-I-188	444	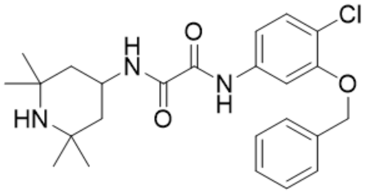
34	TS-I-193	353.8	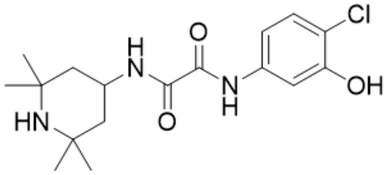
35	TS-I-156	444	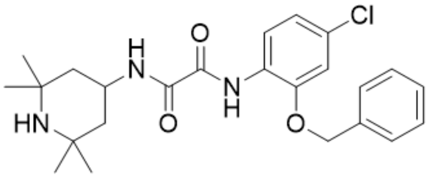
36	TS-I-146	367.9	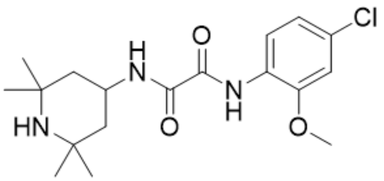

Data S1: Compound Synthesis Procedures

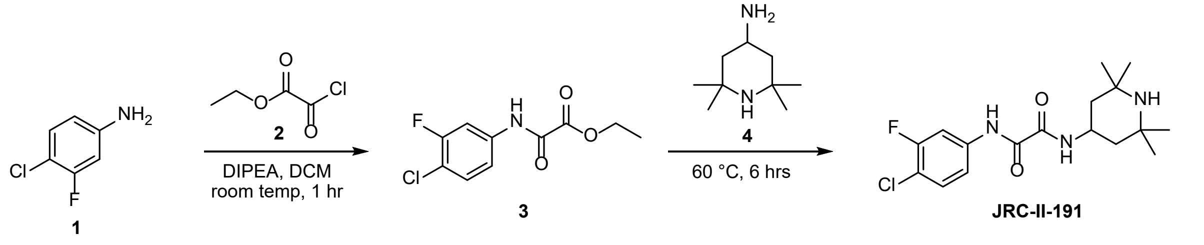

N1-(4-chloro-3-fluorophenyl)-N2-(2,2,6,6-tetramethylpiperidin-4-yl)oxalamide (JRC-II-191): Aniline **(1)** (50 mg, 0.34 mmol, 1.0 equiv), ethyl oxalyl chloride **(2)** (46.91 mg, 0.34 mmol, 1.0 equiv), and diisopropylethylamine (72.5 mg, 0.56 mmol, 1.5 equiv) was dissolved in DCM (0.68 mL) and stirred at room temperature for one hour. Upon completion, amine **(4)** (58.4 mg, 0.374 mmol, 1.1 equiv) was added to the crude mixture and the solution was refluxed at 60 °C for six hours. Upon completion, the reaction was cooled to room temperature and diluted with DCM (0.68 mL) and quenched with water (0.68 mL). This was extracted with DCM (2x 0.68 mL), and the organic extracts were combined and washed with brine (1.36 mL). The combined organic layers were dried with Na_2SO_4, filtered, and concentrated *in vacuo*. Crude reaction mixture was then purified via flash chromatography (2% to 10% EtOAC/hexanes) to yield the title compound in 85% yield. **^{1}H NMR** (500 MHz, $CDCl_3$): 9.30 (s, 1H), 7.70 (dd, J = 10.56, 2.44 Hz, 1H), 7.36 (t, J= 8.46 Hz, 1H), 7.29 (d, J = 8.22 Hz, 1H), 7.22 (dd, J = 8.74, 1.69 Hz, 1H), 4.26 (m, 1H), 1.92 (dd, J = 12.31, 3.69 Hz, 2H), 1.31 – 1.21 (m, 14H) ;**^{13}C NMR** (125 MHz, $CDCl_3$): 158.06 (d, J = 130.1 Hz), 139.22 (d, J = 10.06 Hz), 130.87, 117.23, 117.11, 115.91 (d, J = 3.51 Hz), 108.49, 108.31, 51.13, 44.52, 43.76, 34.73. 29.71, 28.41; **HRMS** (ESI) m/z: $[M+H]^+$ calcd 356.1554, found 356.1551.

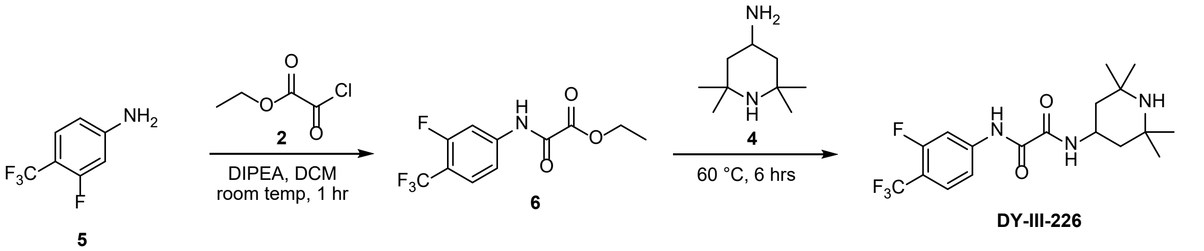

N1-(3-fluoro-4-(trifluoromethyl)phenyl)-N2-(2,2,6,6-tetramethylpiperidin-4-yl)oxalamide (DY-III-226): Aniline **(5)** (50 mg, 0.279 mmol, 1.0 equiv), ethyl oxalyl chloride **(2)** (38.09 mg, 0.279 mmol, 1.0 equiv), and diisopropylethylamine (54.09.5 mg, 0.418 mmol, 1.5 equiv) was dissolved in DCM (0.68 mL) and stirred at room temperature for one hour. Upon completion, amine **(4)** (47.98 mg, 0.307 mmol, 1.1 equiv) was added to the crude mixture and the solution was refluxed at 60 °C for six hours. Upon completion, the reaction was cooled to room temperature and diluted with DCM (0.56 mL) and quenched with water (0.56 mL). This was extracted with DCM (2x 0.56 mL), and the organic extracts were combined and washed with brine (1.16 mL). The combined organic layers were dried with Na_2SO_4, filtered, and concentrated *in vacuo*. Crude reaction mixture was then purified via flash chromatography (2% to 10% EtOAC/hexanes) to yield the title compound in 80% yield. **^{1}H NMR** (500 MHz, $CDCl_3$): 9.48 (s, 1H), 7.75 (dd, J = 12.47, 1.70 Hz, 1H), 7.58 (t, J = 8.10 Hz, 1H), 7.39 (d, J = 8.26 Hz, 1H), 7.36 (dd, J = 8.58, 2.43 Hz), 4.28 (m, 1H), 1.92 (d, J = 12.52, 3.91 Hz, 2H), 1.37 – 1.24 (m, 14H);**^{13}C NMR** (125 MHz, $CDCl_3$):

160.32 (q, J = 252.4 Hz) 158.50, 157.95, 141.27 (d, J = 10.95 Hz), 128.07, 122.52 (q, J = 272. 52, 268.79 Hz), 114.80 (d, 3.12 Hz),108.32, 108.12, 52.89, 43.68, 43.40, 33.76, 29.93, 27.76; **HRMS** (ESI) m/z: $[M+H]^+$ calcd 390.1805, found 390.1803.

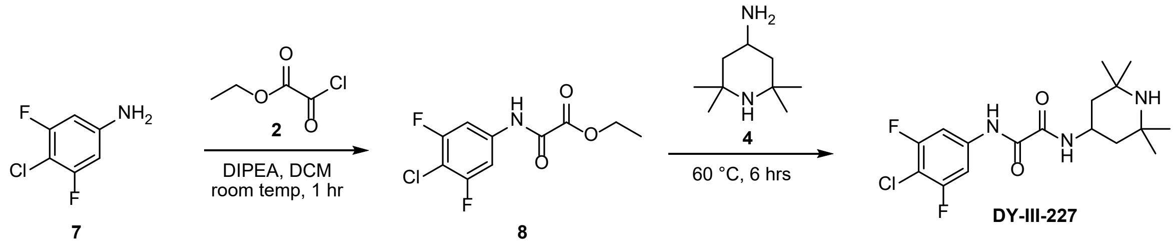

N1-(4-chloro-3,5-difluorophenyl)-N2-(2,2,6,6-tetramethylpiperidin-4-yl)oxalamide (DY-III-227): Aniline **(7)** (50 mg, 0.305 mmol, 1.0 equiv), ethyl oxalyl chloride **(2)** (41.64 mg, 0.305 mmol, 1.0 equiv), and diisopropylethylamine (59.13 mg, 0.457 mmol, 1.5 equiv) was dissolved in DCM (0.61 mL) and stirred at room temperature for one hour. Upon completion, amine **(4)** (52.53 mg, 0.336 mmol, 1.1 equiv) was added to the crude mixture and the solution was refluxed at 60 °C for six hours. Upon completion, the reaction was cooled to room temperature and diluted with DCM (0.61 mL) and quenched with water (0.61 mL). This was extracted with DCM (2x 0.61 mL), and the organic extracts were combined and washed with brine (1.22 mL). The combined organic layers were dried with Na_2SO_4, filtered, and concentrated *in vacuo*. The crude reaction mixture was then purified via flash chromatography (2% to 10% EtOAC/hexanes) to yield the title compound in 83% yield. **^{1}H NMR** (500 MHz, $CDCl_3$): 9.34 (s, 1H), 7.37 (d, J = 8.08 Hz), 7.28 (s, 1H), 4.25 (m, 1H), 1.91 (dd, J = 12.22 3.67 Hz), 1.28 – 1.16 (m, 14H); **^{13}C NMR** (125 MHz, $CDCl_3$): 160.17 (d, J = 5.14 Hz), 158.19 (d, J = 5.14 Hz), 158.14 (d, J = 51.85 Hz), 136.00, 103.97, 103.74, 51.25, 44.64, 43.99, 34.87, 29.95, 28.54 ; **HRMS** (ESI) m/z: $[M+H]^+$ calcd 374.1440, found 374.1432.

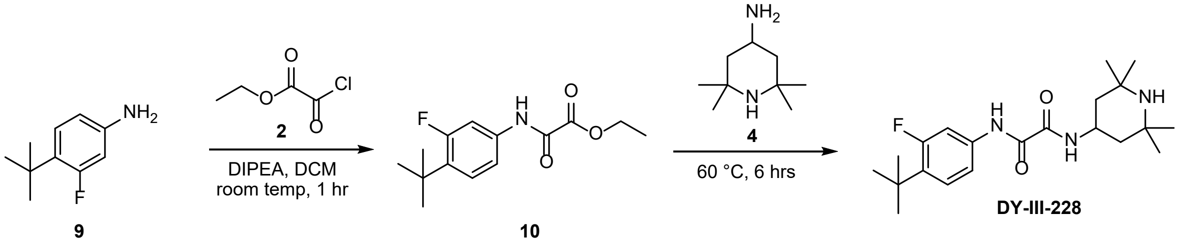

N1-(4-(tert-butyl)-3-fluorophenyl)-N2-(2,2,6,6-tetramethylpiperidin-4-yl)oxalamide (DY-III-228): Aniline **(9)** (50 mg, 0.299 mmol, 1.0 equiv), ethyl oxalyl chloride **(2)** (40.80 mg, 0.299 mmol, 1.0 equiv), and diisopropylethylamine (57.96 mg, 0.448 mmol, 1.5 equiv) was dissolved in DCM (0.61 mL) and stirred at room temperature for one hour. Upon completion, amine **(4)** (46.72 mg, 0.329 mmol, 1.1 equiv) was added to the crude mixture and the solution was refluxed at 60 °C for six hours. Upon completion, the reaction was cooled to room temperature and diluted with DCM (0.60 mL) and quenched with water (0.60 mL). This was extracted with DCM (2x 0.60 mL), and the organic extracts were combined and washed with brine (1.20 mL). The combined organic layers were dried with Na_2SO_4, filtered, and concentrated *in vacuo*. Crude reaction mixture was then purified via flash chromatography (2% to 10% EtOAC/hexanes) to yield the title compound in 89% yield. **^{1}H NMR** (500 MHz, $CDCl_3$): 9.18 (s, 1H), 7.47 (dd, J = 14.22, 2.03 Hz, 1H), 7.43 (d, J = 7.99 Hz), 7.18 (dd, J = 8.69, 1.89 Hz, 1H), 4.29 (m, 1H), 1.94 (dd, J

= 13.34, 3.59 Hz, 2H), 1.38 – 1.25 (m, 14H);^{13}**C NMR** (125 MHz, $CDCl_3$): 159.98 (q, J = 286. 97, 282.66 Hz), 135.40 (d, J = 11.42 Hz), 134.44 (d, J = 12.10 Hz), 127.65, 127.60, 114.77 (d, J = 3.18 Hz), 108.30, 108.11; **HRMS** (ESI) m/z: $[M+H]^+$ calcd 378.2557, found 378.2566.

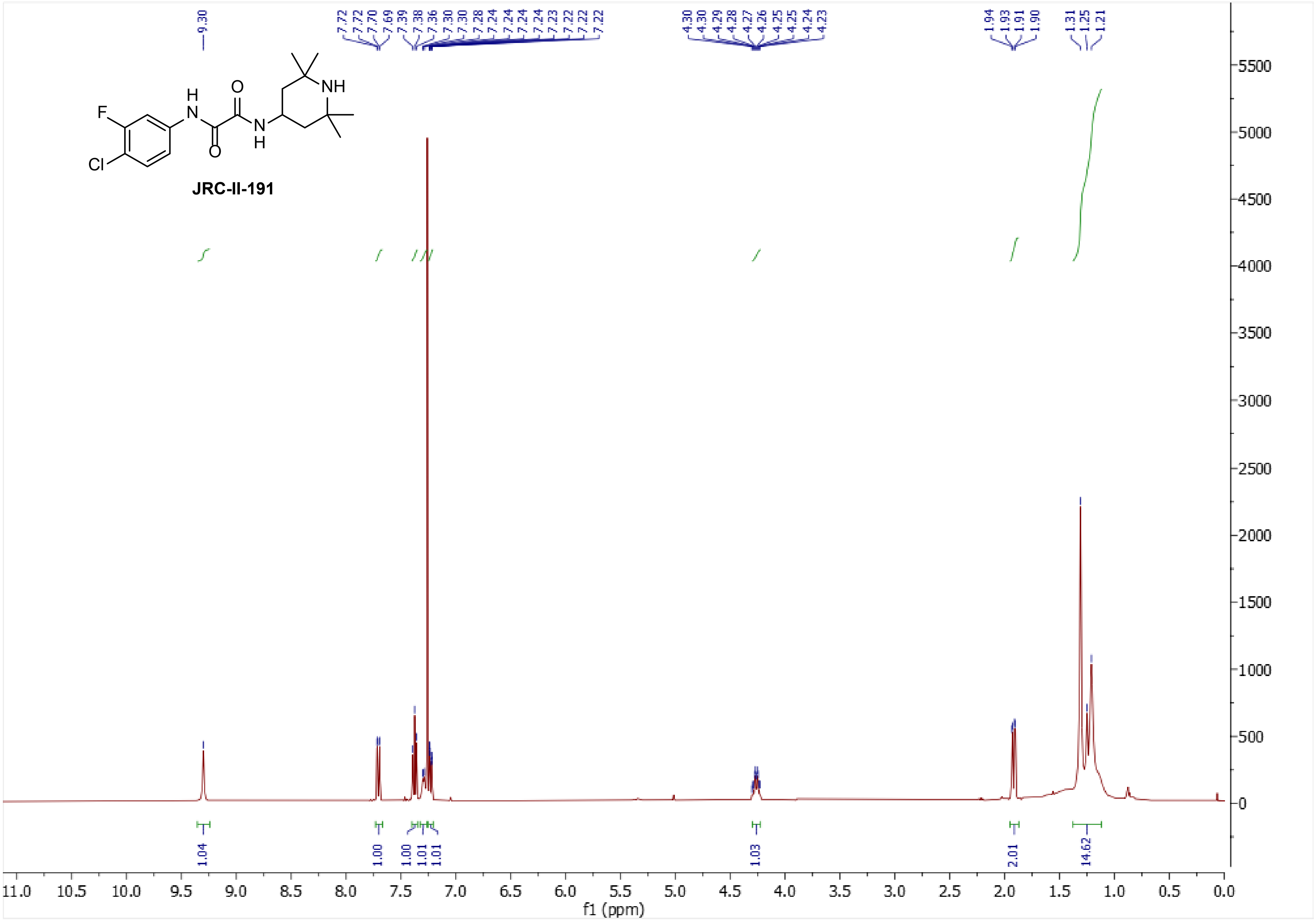

JRC-II-191
9.30
7.72 7.72 7.70 7.69 7.39 7.38 7.36 7.30 7.30 7.28 7.24 7.24 7.24 7.24 7.23 7.22 7.22 7.22
4.30 4.30 4.29 4.28 4.27 4.26 4.25 4.25 4.24 4.23
1.94 1.93 1.91 1.90
1.31 1.25 1.21
1.04 1.00 1.00 1.01 1.01 1.03 2.01 14.62
f1 (ppm)

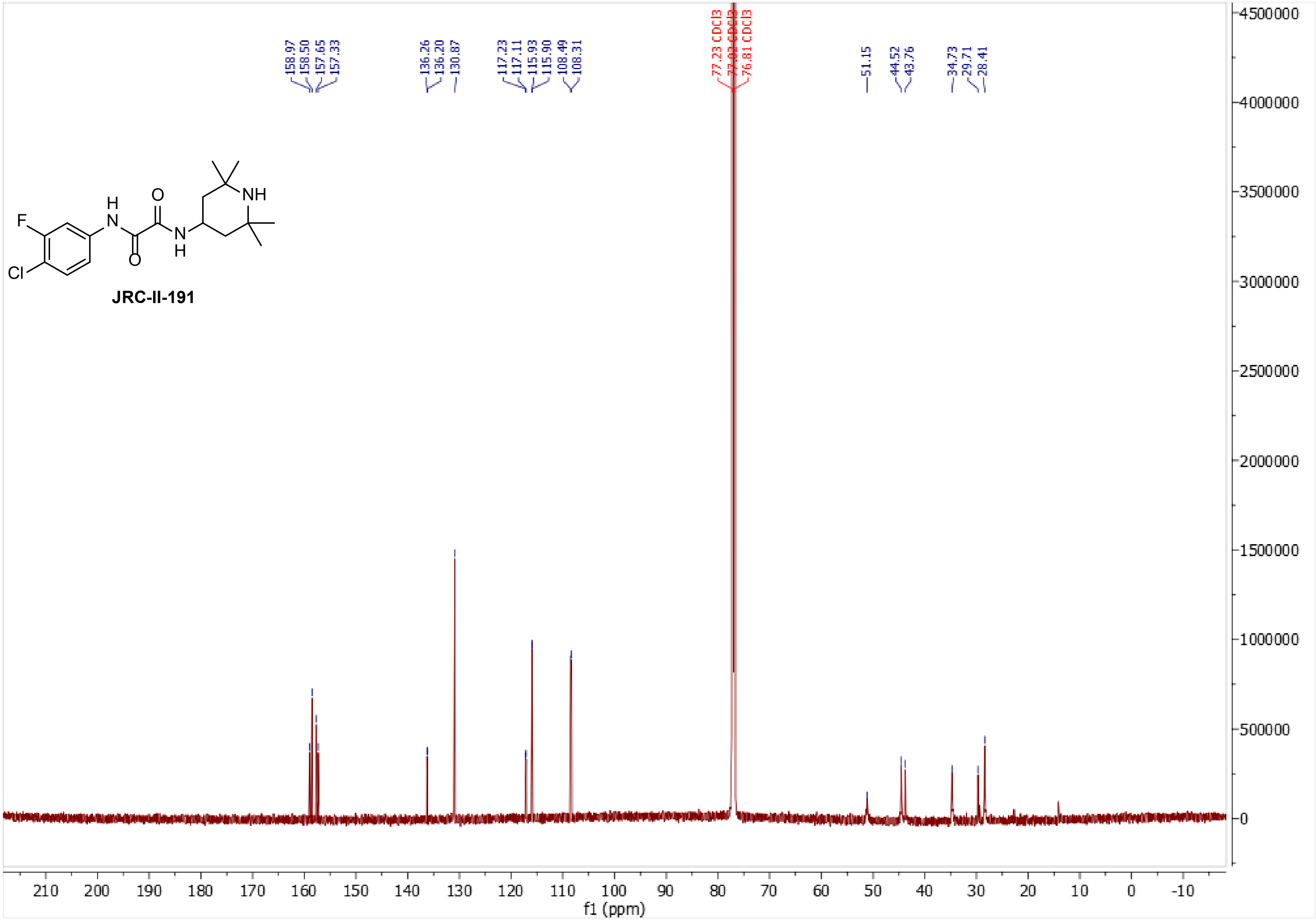

F
Cl
H
N
O
O
N
H
NH
JRC-II-191
158.97
158.50
157.65
157.33
136.26
136.20
130.87
117.23
117.11
115.93
115.90
108.49
108.31
77.23 CDCl3
77.02 CDCl3
76.81 CDCl3
51.15
44.52
43.76
34.73
29.71
28.41
4500000
4000000
3500000
3000000
2500000
2000000
1500000
1000000
500000
0
210 200 190 180 170 160 150 140 130 120 110 100 90 80 70 60 50 40 30 20 10 0 -10
f1 (ppm)

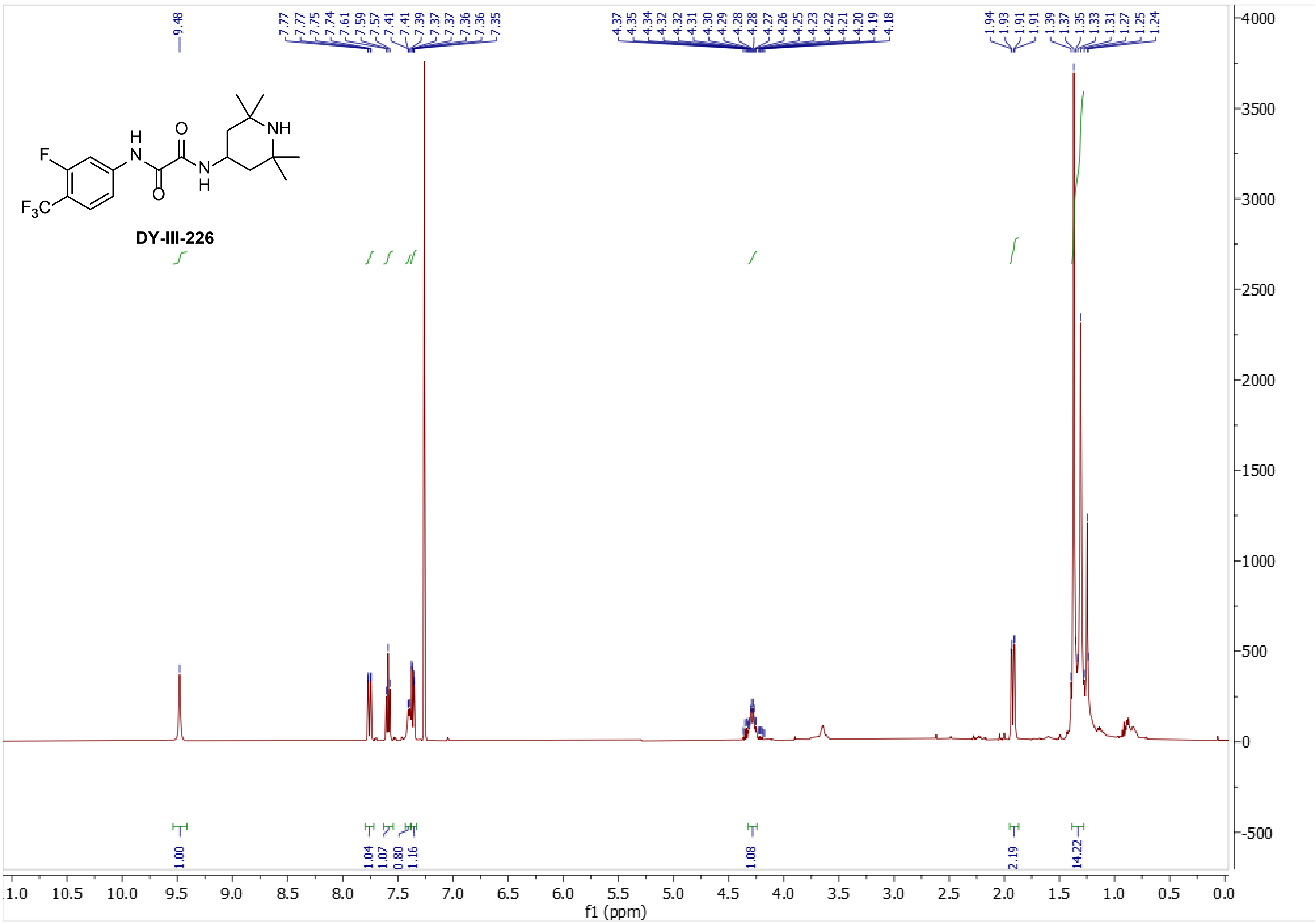

DY-III-226
9.48
7.77 7.77 7.75 7.74 7.61 7.59 7.57 7.41 7.41 7.39 7.37 7.37 7.36 7.36 7.35
4.37 4.35 4.34 4.32 4.32 4.31 4.30 4.29 4.28 4.28 4.27 4.26 4.25 4.23 4.22 4.21 4.20 4.19 4.18
1.94 1.93 1.91 1.91 1.39 1.37 1.35 1.33 1.31 1.27 1.25 1.24
1.00
1.04 1.07 0.80 1.16
1.08
2.19
14.22
f1 (ppm)

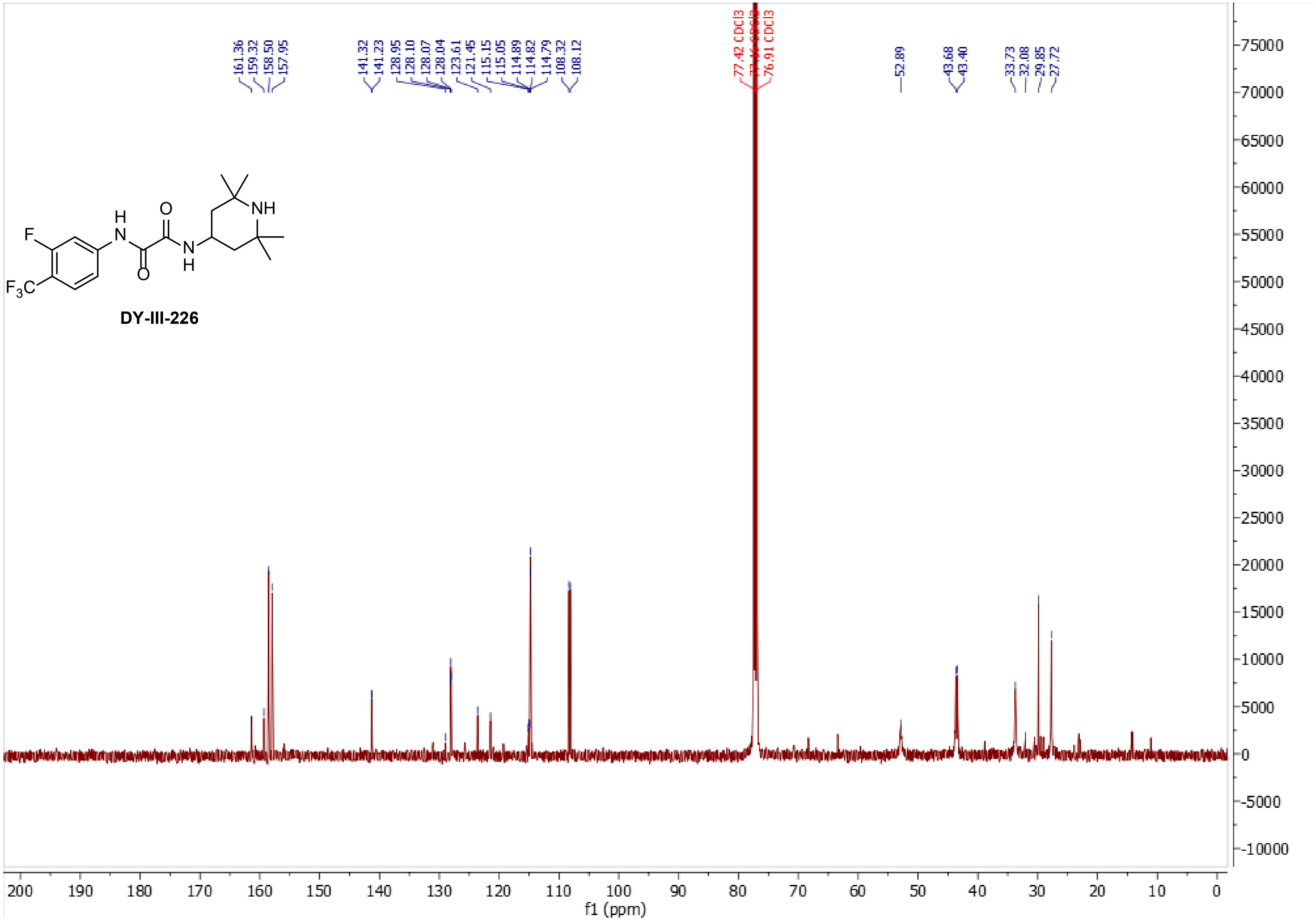

F
F_3C
H
N
O
N
H
NH
DY-III-226
161.36
159.32
158.50
157.95
141.32
141.23
128.95
128.10
128.07
128.04
123.61
121.45
115.15
115.05
114.89
114.82
114.79
108.32
108.12
77.42 CDCl3
76.91 CDCl3
52.89
43.68
43.40
33.73
32.08
29.85
27.72
75000
70000
65000
60000
55000
50000
45000
40000
35000
30000
25000
20000
15000
10000
5000
0
-5000
-10000
200
190
180
170
160
150
140
130
120
110
100
90
80
70
60
50
40
30
20
10
0
f1 (ppm)

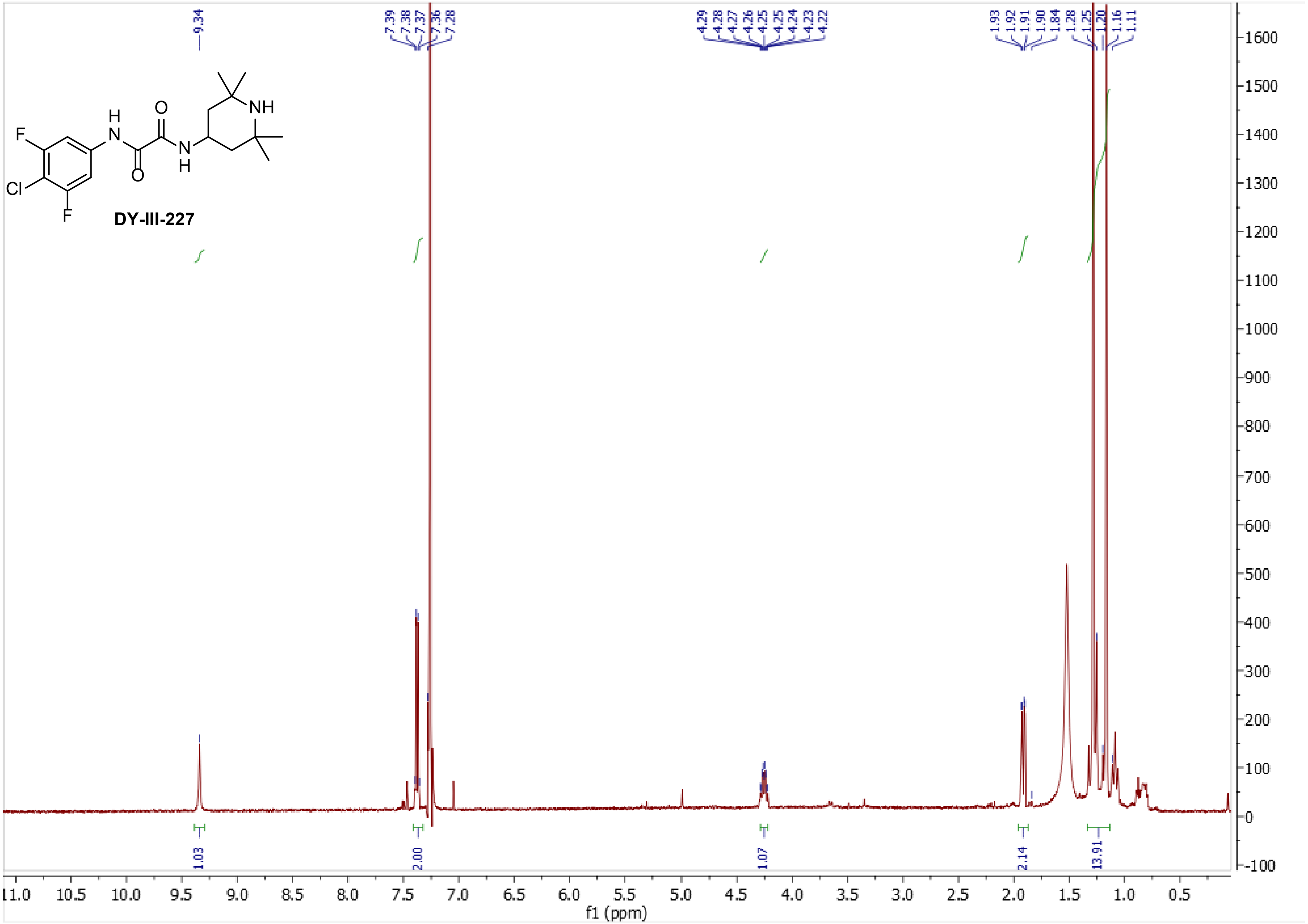
DY-III-227
9.34
7.39
7.38
7.37
7.36
7.28
4.29
4.28
4.27
4.26
4.25
4.25
4.24
4.23
4.22
1.93
1.92
1.91
1.90
1.84
1.28
1.25
1.20
1.16
1.11
1.03
2.00
1.07
2.14
13.91
f1 (ppm)

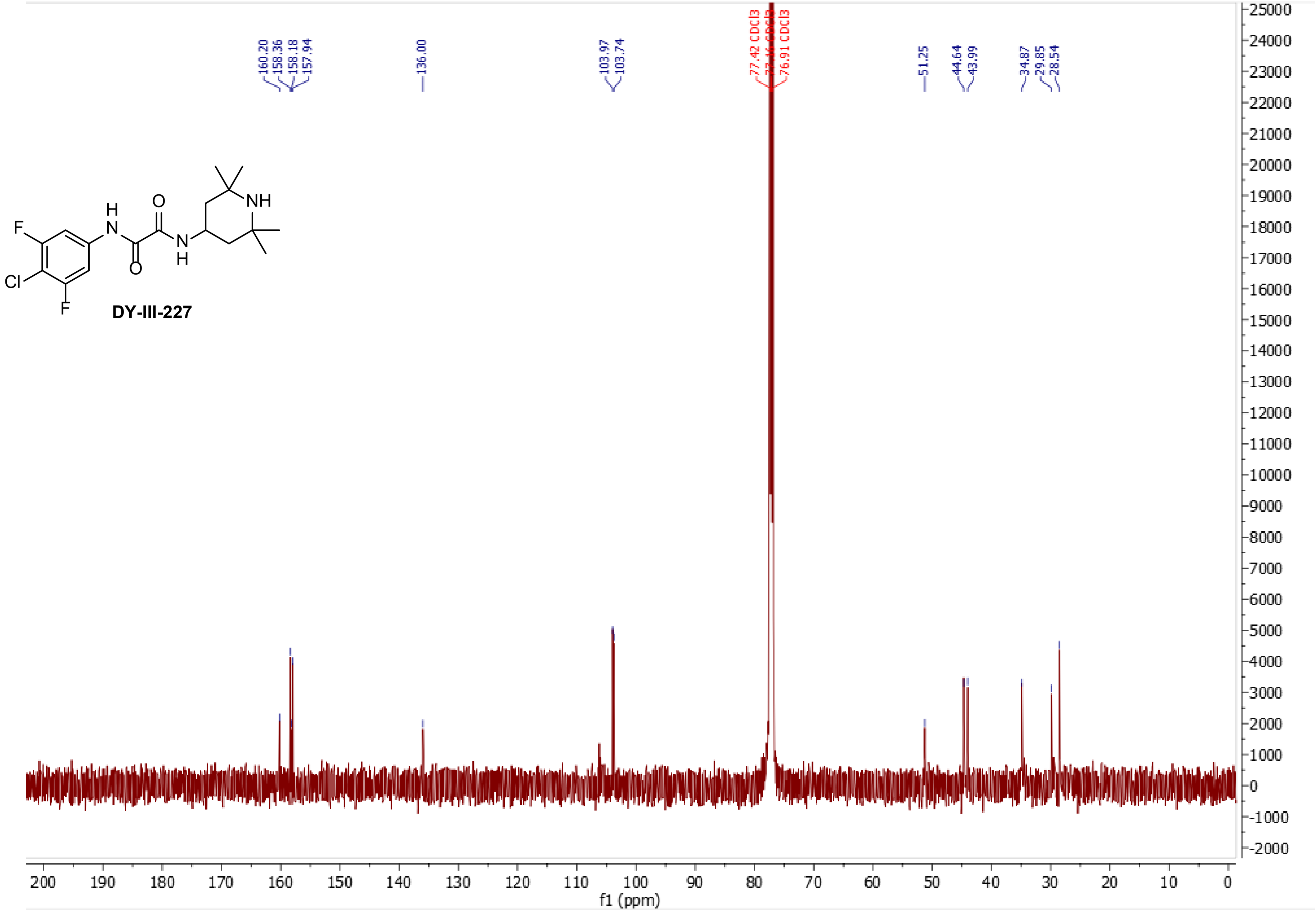

160.20
158.36
158.18
157.94
136.00
103.97
103.74
77.42 CDCl3
76.91 CDCl3
51.25
44.64
43.99
34.87
29.85
28.54
f1 (ppm)

DY-III-227

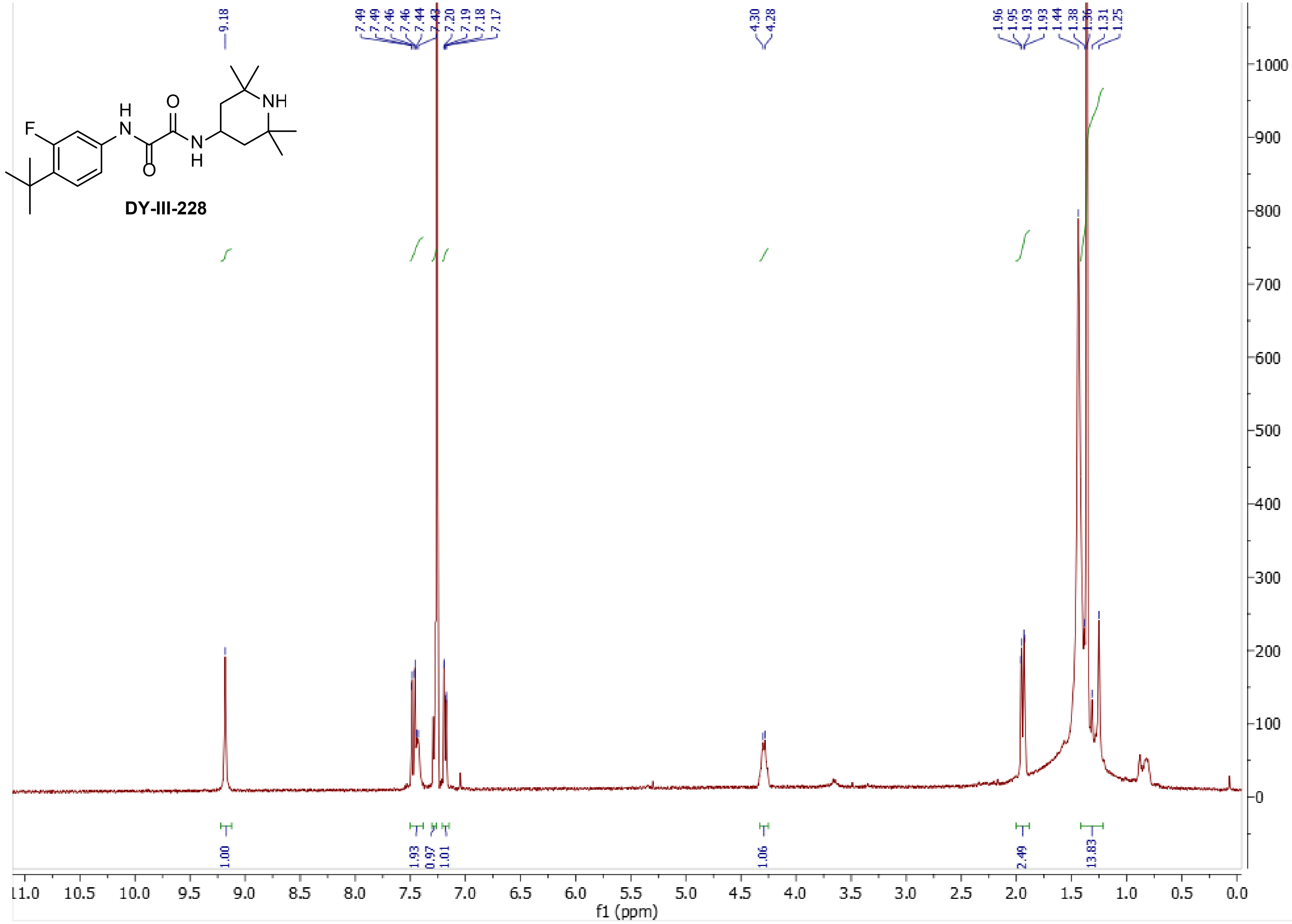

F
H
N
O
O
N
H
NH
DY-III-228
9.18
7.49
7.49
7.46
7.46
7.44
7.43
7.20
7.19
7.18
7.17
4.30
4.28
1.96
1.95
1.93
1.93
1.44
1.38
1.36
1.31
1.25
1.00
1.93
0.97
1.01
1.06
2.49
13.83
f1 (ppm)

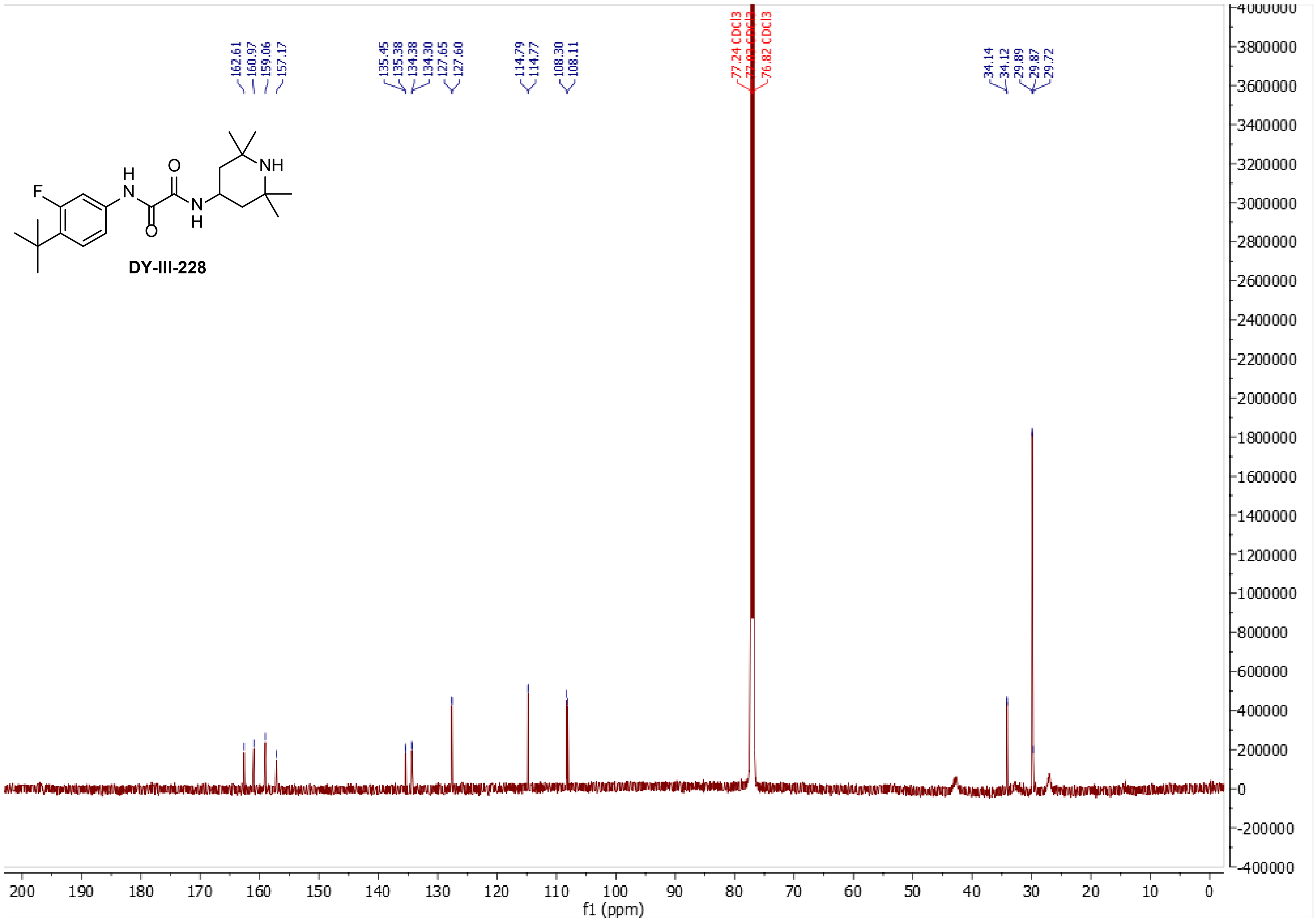
162.61
160.97
159.06
157.17
135.45
135.38
134.38
134.30
127.65
127.60
114.79
114.77
108.30
108.11
77.24 CDCl3
76.82 CDCl3
34.14
34.12
29.89
29.87
29.72
f1 (ppm)

DY-III-228
